# Ecological networks of dissolved organic matter and microorganisms under global change

**DOI:** 10.1038/s41467-022-31251-1

**Published:** 2022-06-23

**Authors:** Ang Hu, Mira Choi, Andrew J. Tanentzap, Jinfu Liu, Kyoung-Soon Jang, Jay T. Lennon, Yongqin Liu, Janne Soininen, Xiancai Lu, Yunlin Zhang, Ji Shen, Jianjun Wang

**Affiliations:** 1 State Key Laboratory of Lake Science and Environment, Nanjing Institute of Geography and Limnology, Chinese Academic of Sciences, Nanjing, 210008 China; 2 College of Resources and Environment, Hunan Agricultural University, Changsha, 410128 China; 3 Bio-Chemical Analysis Team, Korea Basic Science Institute, Cheongju, 28119 South Korea; 4 Ecosystems and Global Change Group, Department of Plant Sciences, University of Cambridge, Cambridge, CB2 3EA UK; 5 Nanchang Institute of Technology, Nanchang, 330099 China; 6 Department of Biology, Indiana University, Bloomington, IN 47405 USA; 7 Center for the Pan-third Pole Environment, Lanzhou University, Lanzhou, 730000 China; 8 State Key Laboratory of Tibetan Plateau Earth System, Resources and Environment, Institute of Tibetan Plateau Research, Chinese Academy of Sciences, Beijing, 100101 China; 9 Department of Geosciences and Geography, University of Helsinki, Helsinki, FIN-00014 Finland; 10 State Key Laboratory for Mineral Deposits Research, School of Earth Sciences and Engineering, Nanjing University, Nanjing, 210093 China; 11 School of Geography and Ocean Science, Nanjing University, Nanjing, 210023 China; 12 University of Chinese Academy of Sciences, Beijing, 100049 China

**Keywords:** Biogeography, Community ecology, Limnology, Biogeochemistry

## Abstract

Microbes regulate the composition and turnover of organic matter. Here we developed a framework called Energy-Diversity-Trait integrative Analysis to quantify how dissolved organic matter and microbes interact along global change drivers of temperature and nutrient enrichment. Negative and positive interactions suggest decomposition and production processes of organic matter, respectively. We applied this framework to manipulative field experiments on mountainsides in subarctic and subtropical climates. In both climates, negative interactions of bipartite networks were more specialized than positive interactions, showing fewer interactions between chemical molecules and bacterial taxa. Nutrient enrichment promoted specialization of positive interactions, but decreased specialization of negative interactions, indicating that organic matter was more vulnerable to decomposition by a greater range of bacteria, particularly at warmer temperatures in the subtropical climate. These two global change drivers influenced specialization of negative interactions most strongly via molecular traits, while molecular traits and bacterial diversity similarly affected specialization of positive interactions.

## Abstract (web summary)

Microbes are intimately linked with the fate of organic matter. Here the authors develop an ecological network framework and show how microbes and dissolved organic matter interact along global change drivers of temperature and nutrient enrichment via manipulative field experiments on mountains.

## Introduction

Dissolved organic matter (DOM) is one of the largest pools of carbon in aquatic ecosystems^1^ and its fate is intimately linked with the metabolism of complex microbial communities^2^. Microbial consortia moderate the diversity of molecules in DOM by degrading larger molecules into smaller molecules and by synthesizing more refractory compounds from labile substrates^3,4^. Together, these metabolic processes lead to the emergence of molecular traits (Box 1), such as molecular weight, chemical structure, stoichiometry, oxidation state, and bioavailability^5–7^, all of which have consequences for the fate and persistence of DOM^8,9^. Because it serves as an energy and carbon source for metabolism, DOM also influences the diversity, structure, and function of microbial communities^10–12^. The resulting associations between DOM and microbes can now be characterised in both aquatic^13–15^ and terrestrial^16^ ecosystems owing to recent advances in ultrahigh-resolution mass spectrometry and high-throughput sequencing. Ultrahigh-resolution mass spectrometry specifically identifies individual molecular formulae within DOM pools. This approach provides more information on the diversity and traits of DOM than available bulk measurements, like those based on absorbance and fluorescence spectroscopy, which are generally lacking in resolution. Despite these technological developments, challenges remain about how DOM-microbe associations can be quantified in nature, and are interactively and independently affected by global environmental change through human-induced perturbations leading to elevated temperatures and nutrient enrichment.

**Fig. 1:**
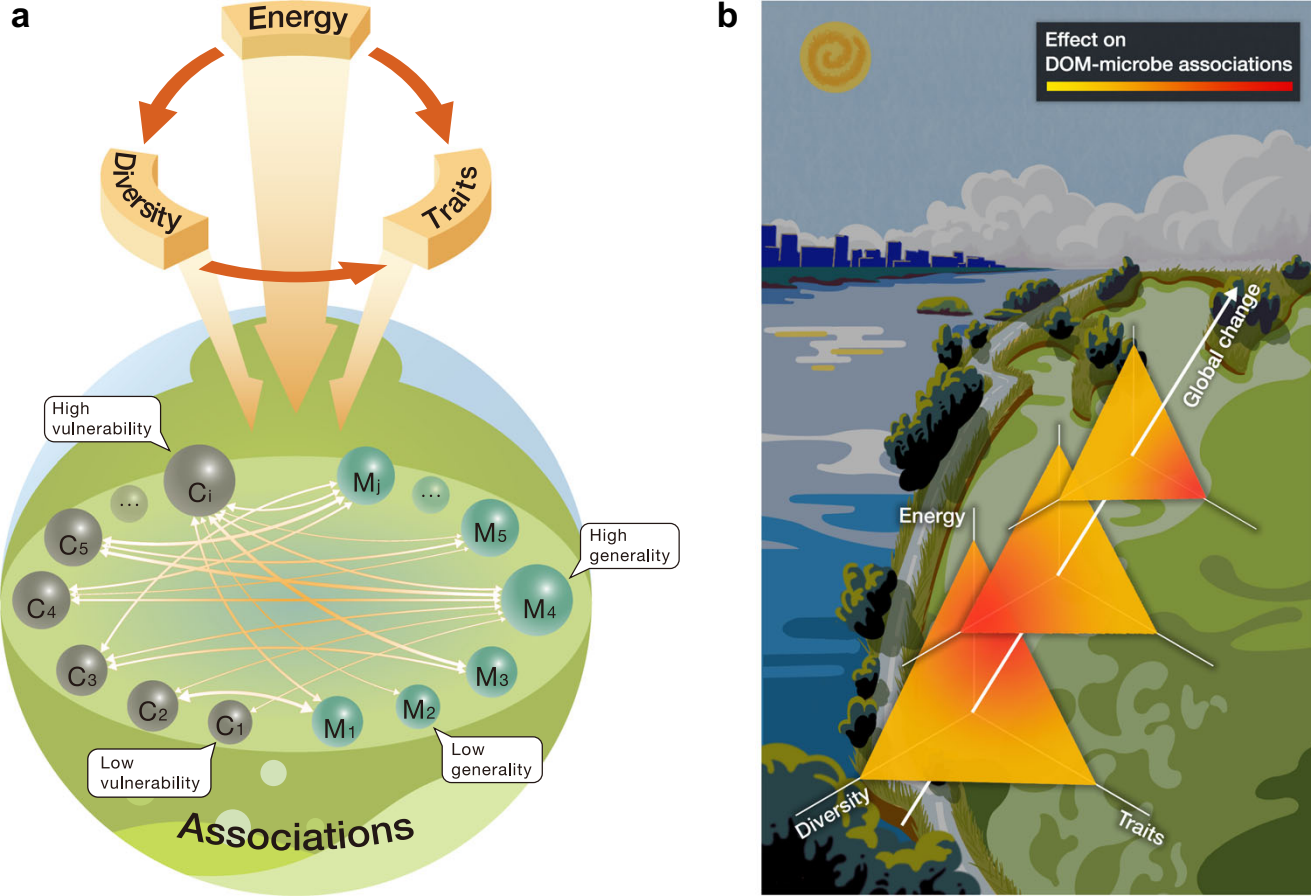
A framework for studying the effects of global change on DOM-microbe associations. **a** DOM-microbe associations are affected by the three proximal drivers, namely energy supply and both the diversity and traits of DOM and microbes. The relationships among the three drivers and their influences on the associations are shown with single-sided arrows. The DOM-microbe associations, indicated by double-sided arrows, are measured by bipartite interactions between DOM molecules (circles C_1_–C_i_) and microbial taxa (circles M_1_–M_j_). The size of circles indicates the abundance of DOM molecules or microbial species, and the width of arrows is the magnitude of associations. Commonly used indices summarise the specialization of individual molecule *i* and microbial species *j*, which describes the levels of “vulnerability” of DOM molecules and “generality” of microbial species. **b** Conceptual framework for understanding DOM-microbe associations under distal drivers such as global change via the three proximal drivers. For better 3D visualization, the sizes of triangles decrease towards the top-right, and the colour changes towards different corners of the triangles represent variations in the relative importance of different proximal drivers under a global change scenario. The background depicts the primary motivation of this study in examining distal drivers of climate change and eutrophication in Taihu Lake, China. The left and right waters indicate clean and cyanobacteria-dominated lake states, respectively, and are separated by a road having the shapes of western lakeshore and northern Zhushan and Meiliang Bays of Taihu Lake. We established field microcosms on mountainsides by adding sediments collected from the lake centre, and designed nutrient levels and N/P ratio based on nutrient conditions of this lake^29^.

The effects of global change on DOM-microbe associations can be viewed through three proximal drivers (Fig. 1). First, energy supply, such as primary productivity, represents the major input of DOM that fuels microbial metabolism^17,18^. Elevated temperature and nutrient inputs often stimulate primary productivity in ways that influence the composition and availability of organic matter^19,20^. For instance, microbial decomposition of DOM can be limited by substrate and nutrient availability that provide sufficient energy and material for synthesis of requisite extracellular and transport enzymes^21,22^. Second, DOM-microbe associations are affected by diversity. For example, an increase in the diversity of DOM can promote microbial diversity, and vice versa^14^. Such patterns may arise because resource diversity promotes microbial specialization during biochemical transformations by creating more unique resource niches for consumers to partition^23,24^. Likewise, higher microbial diversity can provide more metabolic pathways (but not always elevated activity) to decompose and produce molecules, which can influence the vulnerability of DOM to degradation^3^. Third, DOM-microbe associations depend on the molecular traits of DOM, such as its bioavailability, measured by chemical proxies such as H/C ratios of individual molecules^25^, as well as life-history traits of microbes (e.g., *r*-selected copiotrophs versus *K*-selected oligotrophs^26^ or resource generalists versus resource specialists^24^).

**Fig. 2:**
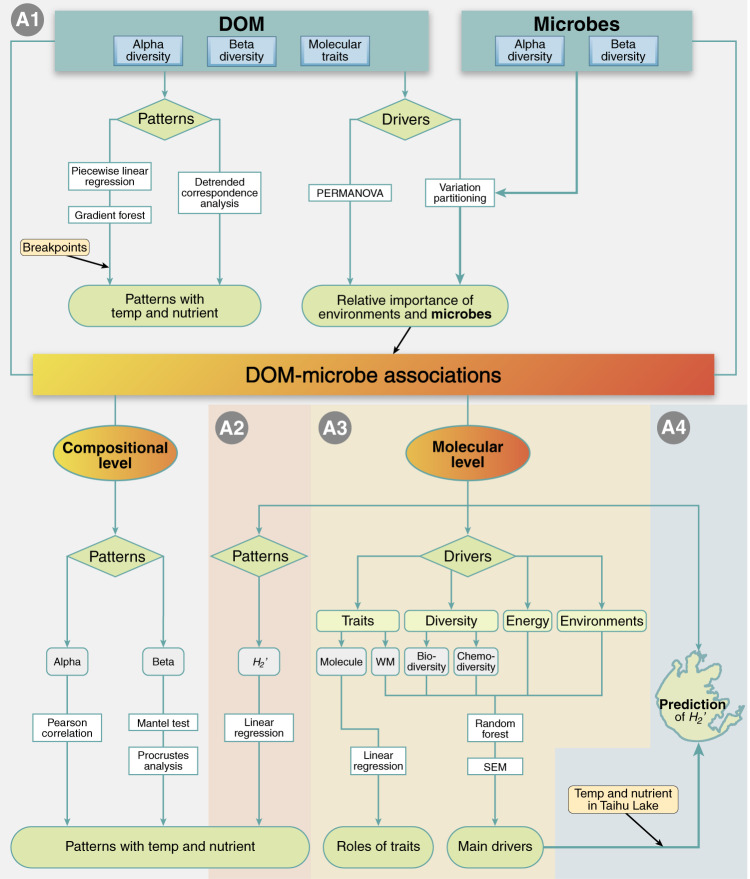
Outline of statistical analyses to address the study aims. A1–A3 refer to the three main questions stated in the ‘Introduction’, and A4 refers to the prediction of *H*_2_′ in Taihu Lake. The response or explanatory variables in grey or light blue boxes were examined by the statistical methods in white boxes for the main purposes indicated in light green boxes or an irregular shape box. The irregular shape shows the lakeshore of Taihu Lake. PERMANOVA: permutational multivariate analysis of variance. SEM: structural equation model. Alpha: alpha-diversity of DOM and microbes. Beta: beta-diversity of either DOM or microbes. WM: weighted means of formula-based molecular traits. Temp: temperature. Details for each statistical analysis such as main purposes were described in the ‘Methods’.

To examine how DOM-microbe associations vary under global change because of the three aforementioned proximal drivers, we developed a framework called Energy-Diversity-Trait integrative Analysis (EDTiA) (Fig. 1). The first step of EDTiA involves the construction of bipartite networks^27^ to quantify the degree of specialization between organic molecules and microbial taxa (Box 1). In the DOM-microbe networks, individual DOM molecules are only connected to microbial taxa that use that specific molecule, while the direct interactions within molecules or taxa are not explicitly considered. The second step of EDTiA involves the investigation of ecological networks using an interaction specialization metric, *H*_2_′, which is derived from Shannon index^28^ (Box 1). By taking resource-consumer relationships among a collection of samples into account, an elevated *H*_2_′ means that there is a high degree of specialization between DOM and microbes^28^, where in the extreme example, one bacterial taxon consumes or produces a single DOM molecule. By contrast, lower *H*_2_′ values reflect a more generalized bipartite network where different DOM molecules can be used by a large number of bacterial taxa. The third step of EDTiA provides a statistical-based means for assessing the relative importance of global change on the specialization of DOM-microbe associations via the three proximal drivers (Fig. 2).

In this study, we developed and used the EDTiA framework to evaluate how DOM-microbe associations were independently and jointly influenced by temperature and nutrient enrichment (Figs. 1, 2) in a manipulative field experiment on two mountainsides, a subtropical one in China and a subarctic one in Norway^29^. This approach involved creating microcosms with consistent initial DOM composition but different locally colonised microbial communities and newly produced endogenous DOM. Such field experiments along natural climate gradients can disentangle temperature effects on DOM-microbe associations from nutrient enrichment, unlike in natural ecosystems where these drivers are typically confounded. The framework explicitly quantifies the relative importance of the direct effects of temperature and nutrient enrichment and the indirect effects via the proximal drivers of energy, diversity, and traits. Briefly, we selected five different elevations on each mountainside, and at each elevation, we established 30 microcosms composed of natural lake sediments and artificial lake water with ten different nutrient levels. The nutrient levels were selected based on conditions of Taihu Lake, a large eutrophic shallow lake in China with an area of 2338 km^2^. The sediments originated from Taihu Lake and were added to each microcosm after sterilisation to ensure identical initial DOM supply and composition. Microcosms were incubated in the field for one month allowing airborne bacteria to colonise, and sediment bacteria were examined using high-throughput sequencing of 16S rRNA genes^29^. In addition, we applied ultrahigh-resolution electrospray ionization Fourier transform ion cyclotron resonance mass spectrometry (FT-ICR MS) to examine sediment DOM features, such as the diversity of molecular formulas (hereafter chemodiversity) and molecular traits (Table S1).

With this experiment, we address three main questions: (1) at a compositional-level, how does DOM molecular composition and its association with microbial biodiversity vary along temperature and nutrient gradients?, (2) at a molecular-level, how does the degree of specialization between DOM and microbes estimated from interaction networks vary along temperature and nutrient gradients? and (3) how is DOM-microbe specialization interactively and independently influenced by temperature and nutrient enrichment via the three proximal drivers of energy, diversity, and traits? We then discuss how the effects of these drivers could be used to predict the spatiotemporal changes in specialization elsewhere under different temperature and nutrient scenarios. We focus on Taihu Lake as case study given its socioeconomic and cultural importance with over 60 million people living in the Taihu Basin, and because it is where our experimental sediments and nutrient reference conditions originated from. Together, our study advances biogeochemical modelling and improves predictions about both carbon turnover and resource-based feedbacks on microbial diversity.

Box 1 Glossary of terms for merging organic geochemistry and ecology *Molecular composition:* The chemical composition of dissolved organic matter (DOM) that is a complex mixture of reduced carbon compounds bound to heteroatoms such as oxygen, nitrogen, phosphorus, and sulphur. The composition is characterised by the identity and intensity of molecular formulae in a sample.

*Molecular trait:* The structural features of an individual DOM molecule deduced from molecular formula that can be assigned precisely from ultrahigh resolution mass spectrometry. These molecular-level traits include molecular weight, stoichiometry, chemical structure, oxidation state and bioavailability (e.g., lability and recalcitrance), and can be upscaled to an entire sample (i.e., compositional-level) with weighted means.

*DOM compound classes:* The groupings of DOM molecules that are categorised based on molecular traits such as H/C and O/C ratios: lipids, proteins, amino sugars, carbohydrates, unsaturated hydrocarbons, lignin, tannin, and condensed aromatics.

*DOM-microbe associations:* The relationships between DOM and microbes at multiple levels, including the compositional-level and species/molecular-level. Compositional-level associations involve correlations of alpha or beta diversity between DOM and microbes. Species/molecular-level associations refer to interactions between individual DOM molecules and microbial taxa, which can be upscaled to an interaction network by network properties, such as the measures of specialization.

*DOM-microbe bipartite network:* The interaction network between DOM and microbes, where individual organic molecules are only connected to microbial taxa that may consume or produce that specific molecule, while the direct interactions within molecules or taxa are not explicitly considered^27^. The network is a species/molecular-level means to quantify DOM-microbe associations and consists of either positive or negative interaction networks that are inferred by the positive and negative coefficients of correlation analyses such as SparCC^35^.

*DOM-microbe specialization:* A network property describing how much DOM molecules and microbial taxa interact with each other in a bipartite ecological network, that is the levels of “vulnerability” of DOM molecules and “generality” of microbial taxa. Specialization could be quantified by the standardised Shannon index *H*_2_′^28^ that considers the interaction frequency and strength between the two network parties. Elevated *H*_2_′ values indicate a high degree of specialization, while lower values suggest increased generalization, that is, higher vulnerability of DOM and/or higher generality of microbes (Fig. 1a).

## Results and discussion

### DOM features and their microbial associations at a compositional-level

**Fig. 3:**
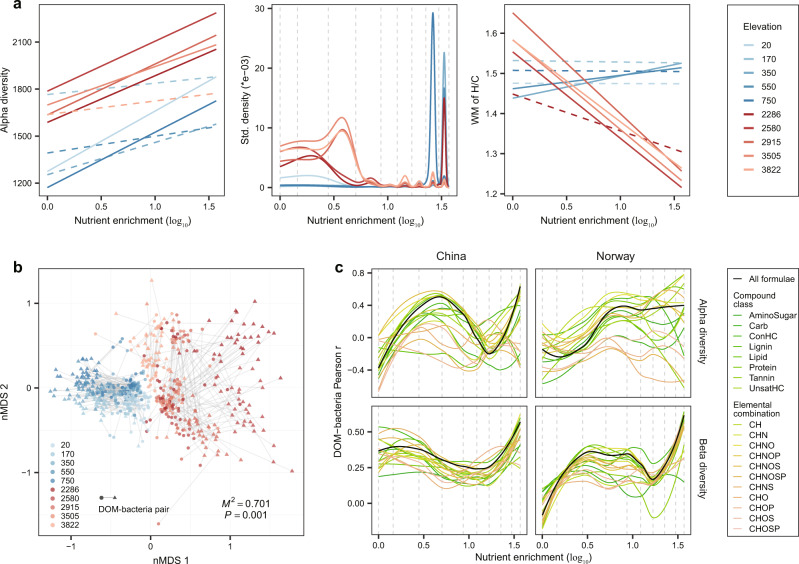
DOM features and their bacterial associations at a compositional level. **a** The effects of nutrient enrichment on DOM alpha diversity (richness), composition and molecular traits (e.g., H/C ratio) for all formulae across different elevations in China (red lines, with lower elevations in more red colours) and Norway (blue lines, with higher elevations in more blue colours). We used nitrate addition to represent nutrient enrichment as the ratio between nitrate and phosphorus in the initial overlying water was constant. Molecular richness and weighted mean (WM) of H/C ratio were plotted against the nutrient gradient of nitrate, and their relationships are indicated by solid (*P* ≤ 0.05) or dotted (*P* > 0.05) lines estimated using linear models with one-sided F-statistics. For better visualization, we did not include the data points in Fig. 3a but showed detailed scatter plots and statistics in Fig. S3. To visualise the DOM compositional changes, we plotted the distribution of breakpoints of each molecule regarding its abundance occurring along the nutrient gradient with gradient forest analysis^30^. **b** The congruence between DOM and bacterial compositions across different elevations in China and Norway was examined using Procrustes analysis^32,33^. The NMDS plots the position of DOM and bacterial assemblages that have been optimized based on Procrustes analysis. Each line with circle and triangle ends connects to a single community of DOM and bacteria, respectively, and is coloured by elevation in either China (red) or Norway (blue). The fit of overall Procrustes transformation is reported as the *M*^2^ value, and the significance was examined using a permutation-based approach with 999 permutations and the two-sided statistically significant *P* value is shown. **c** The effects of nutrient enrichment on DOM-bacteria associations. The associations were quantified by the Pearson correlation coefficient *r* between alpha diversity of DOM and bacteria (upper panel), and by the Mantel correlation *r* between the beta diversity of DOM and bacteria (lower panel). We then visualised correlation *r* values with loess regression models along the nutrient gradient, and these correlations at each nutrient level are shown in Fig. S8. The colours of the lines indicate the DOM composition for all formulae and categories of compound classes or elemental combinations.

The diversity and molecular traits of DOM were strongly controlled by nutrient enrichment, and to a lesser by temperature (that is, elevation), on both subtropical and subarctic mountainsides (Figs. S1, S2). Nutrient enrichment generally increased alpha diversity (i.e., molecular richness) of DOM across all elevations when all molecular formulae were considered (Figs. 3a, S3). We identified abrupt changes in molecular composition along the nutrient gradient that mostly occurred between 1.80 and 4.05 mg N L^−1^ for all molecules at each elevation using both gradient forest analysis^30^ (Fig. 3a) and piecewise regression^31^ (Fig. S4). The effects of nutrient enrichment on molecular traits, however, varied between the two ecoregions (Figs. 3a, S3, S5). For instance, the weighted mean of the H/C ratio in each microcosm decreased with nutrient addition to <1.5, especially at high elevations in China, indicating less bioavailable DOM (Figs. 3a, S3c). The ratio remained consistently higher (≥1.5) across all nutrient levels in Norway (Figs. 3a, S3c). Given that the initial DOM composition in our study was identical everywhere, this finding suggests that the contrasting responses reflect differences in the temperature sensitivity of decomposition and/or nutrient-limited production of DOM by colonising microbes. This inability to resolve the mechanisms underlying these patterns further highlights the need for the EDTiA framework. EDTiA can help disentangle underlying drivers by explicitly quantifying the direct and indirect effects of temperature and nutrient enrichment on the associations between DOM and microbes via intermediate environmental variables such as energy, diversity and traits (Fig. 1).

DOM composition was strongly associated with bacterial communities in both regions, and was mediated by temperature and nutrient enrichment. For instance, although environment (temperature and nutrients) and energy supply had dominant effects on DOM composition, their shared effects with biodiversity (2.7–13.1% of explained variation) indicated that these variables also indirectly influenced the associations between DOM and bacteria (Fig. S6). These DOM-bacteria associations were also supported by the fact that DOM composition was significantly predicted from bacterial community with Procrustes analysis^32,33^ (*M*^2^ = 0.701, *P* ≤ 0.001; Fig. 3b), while their associations varied with temperature and nutrient enrichment. For example, compositional differences, indicated by the residuals of Procrustes analysis, significantly (*P* ≤ 0.05) decreased for all compound classes or elemental combinations at colder temperatures in China (Fig. S7). In Norway, the differences were always lower, on average, and did not vary with temperature (Fig. S7). Nutrient enrichment influenced the correlations between the number (“alpha diversity”) of DOM molecules and bacterial operational taxonomic units (OTUs) in each microcosm, or between the similarity in composition (“beta diversity”) of DOM and bacterial communities for all pairwise combinations of microcosms within a region (Fig. 3c). These results were consistent for individual compound classes or elemental combinations (Fig. 3c). Interestingly, the coordinated compositional changes in DOM and bacteria, measured by the Mantel correlation^34^ between their beta diversities among pairwise microcosms, increased more strongly with nutrient enrichment in Norway than in China, especially at low nutrient levels beneath 1.80 mg N L^−1^ (Figs. 3c, S8).

### Ecological networks between DOM and bacteria at a molecular-level

**Fig. 4:**
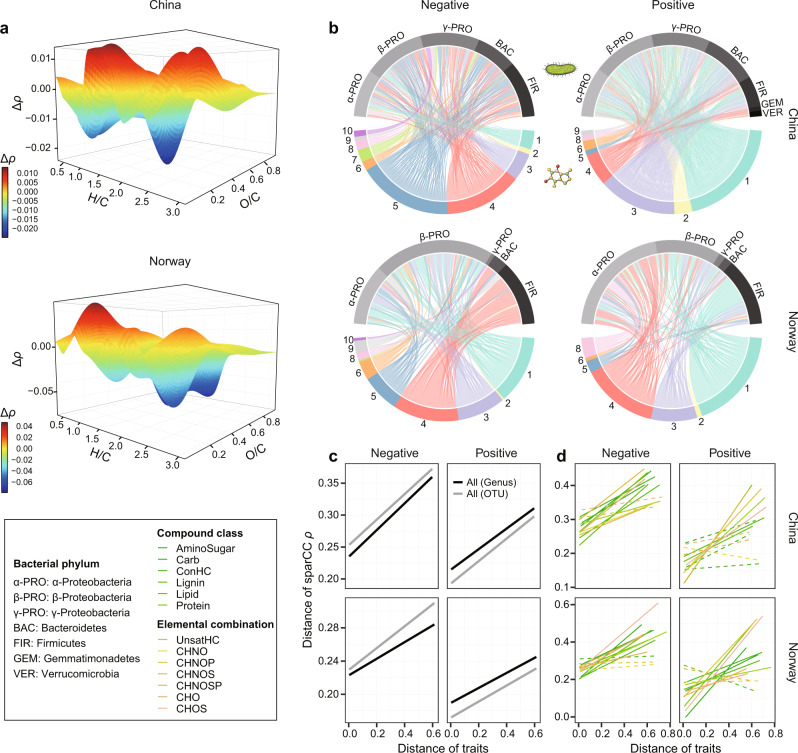
Ecological networks between DOM and bacteria. **a** Strength of the correlations between DOM molecules and bacterial OTUs in China (upper panel) and Norway (lower panel). For each molecule across the 150 samples in each region, we subtracted the mean absolute Spearman’s rank correlation coefficient *ρ* of all the negative correlations with individual bacterial OTUs from the mean of the positive correlations to derive Δ*ρ*. Δ*ρ* was further visualised against the molecular traits H/C and O/C. **b** The negative (left panel) and positive (right panel) bipartite networks between DOM molecules and bacterial genera in China (upper panel) or Norway (lower panel) estimated using SparCC (Sparse Correlations for Compositional data)^35^. Upper nodes represent bacterial genera coloured by their phylum, while lower nodes represent DOM molecules coloured by the ten clusters obtained with hierarchical cluster analysis based on 16 molecular traits described in Fig. S12a and Table S1. The traits of H/C and O/C for molecules in each cluster are shown in Fig. S12b. A line connecting two nodes indicates an interaction between a DOM molecule and bacterial genus. The ten clusters are indicated by the numbers below the lower nodes. **c**, **d** We examined the relationships between molecular traits and the negative (left panel) or positive (right panel) DOM-bacteria bipartite networks in China (upper panel) or Norway (lower panel). Between all pairs of DOM molecules, we calculated pairwise Gower distances of their molecular traits (that is, distance of traits), or the SparCC *ρ* of molecules with bacterial OTUs (that is, distance of SparCC *ρ*). Statistical significance between these two distance matrices was determined with a two-sided Mantel test with 999 permutations and indicated by solid (*P* ≤ 0.05) or dotted (*P* > 0.05) lines. We considered all formulae (**c**) and also subsets of formulae within the category of compound classes or elemental combinations (**d**). For all formulae (**c**), we calculated SparCC correlation coefficients based on both bacterial OTUs (grey lines) and genera (black lines).

To quantify the associations between DOM and bacteria further from a molecular-level perspective, we first correlated the relative abundance of each DOM molecule and bacterial taxa. According to resource-consumer relationships, the negative network interactions inferred by negative correlations likely indicate the degradation of larger molecules into smaller structures, while the positive interactions may relate to the production of new molecules via degradation or biosynthetic processes. We found that the distribution of negative and positive Spearman correlations between DOM molecules and bacterial OTUs depended strongly on molecular traits. For example, molecules that were more labile, such as those with H/C ≥ 1.5, were more likely to have negative correlation coefficients (*ρ*) with individual OTUs (*P* ≤ 0.05). In contrast, molecules that were more recalcitrant (H/C < 1.5) generally showed more positive correlations (*P* ≤ 0.05), especially in Norway (Fig. S9). These findings were even more clearly supported by the differences between the mean of the positive and negative *ρ* values for each molecule (Figs. 4a, S10). Correlations with individual OTUs were predominantly negative for molecules within a H/C of 1.5–2.0 and O/C of 0.4–1.0, suggesting they are largely the reactants of degradation processes, while *ρ* differences peaked with mainly positive values at a H/C of 1.0–1.5 and O/C of 0–0.5 indicative of in situ production (Fig. 4a).

Subsequently, we quantified DOM-bacteria interactions along temperature and nutrient gradients using the EDTiA framework. We built bipartite networks of negative and positive interactions between DOM and bacteria at the genus level using Sparse Correlations for Compositional data (SparCC)^35^. SparCC can infer interaction strength by statistical correlations with a high degree of accuracy for sparse matrices, i.e. where there are many 0’s^35^. In total, there were 6916 and 8409 interactions for negative and positive networks (|SparCC *ρ* | ≥ 0.3), respectively, in China, and 1313 and 2888 negative and positive interactions, respectively, in Norway (Fig. 4b). The weighted mean of the percentage of SparCC *ρ* values that were strongly negative (*P* ≤ 0.05) increased towards high nutrient levels almost exclusively in China (Fig. S11). The weighted mean of negative SparCC *ρ* also increased with nutrient enrichment in China (Fig. S11) and these results together suggest that nutrient enrichment increased both the number and strength of negative interactions.

We found that the negative and positive interactions strongly depended on molecular traits, which was further supported by three observations of bipartite networks. First, the negative and positive interactions were associated with different molecule groups, as categorised by a hierarchical cluster analysis of 16 molecular traits (Figs. 4b, S12). This cluster analysis identified two groups of molecules (clusters 4 and 5) that were largely recalcitrant with a H/C of <1.5, while two separate groups (clusters 1 and 3) mostly included labile molecules with a H/C of ≥1.5 (Fig. S12). In China, negative interactions were dominant between the molecules in clusters 4 or 5 and the bacteria in phyla Proteobacteria, Bacteroidetes, or Firmicutes, while the positive interactions were mostly linked to the molecule clusters 1 and 3 (Fig. 4b). In Norway, the molecule cluster 4 was mainly negatively linked to Firmicutes and positively linked to α- and β-Proteobacteria (Fig. 4b). Second, molecules generally covaried more similarly with bacteria as they were more similar in their traits. This was indicated by the positive linear slopes between the pairwise differences in both the traits of DOM molecules and their interaction strength measured by SparCC *ρ* values (Mantel test, *P* ≤ 0.001 in each region; Fig. 4c). For example, had DOM molecules more similar H/C or O/C values, they correlated more similarly with bacterial taxa. Third, molecular traits were more strongly correlated with the strength of DOM-bacteria interactions in the negative than positive interaction networks for all molecules (Fig. 4c), which was also true for most of the networks when considering compound classes or elemental combinations (Fig. 4d). These correlations, consistent at both the genus and OTU levels (Fig. 4c), indicate that molecular traits may be better at predicting the decomposition than production of DOM.

**Fig. 5:**
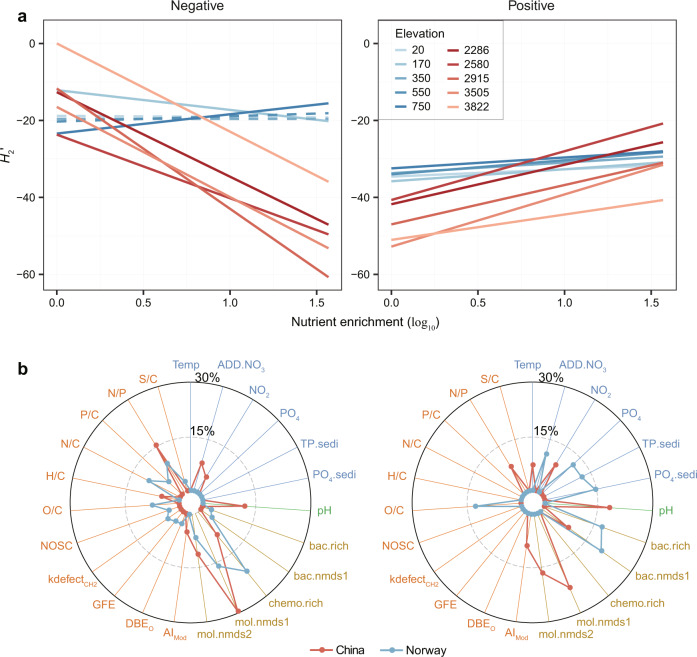
Relative importance of diversity and molecular traits in explaining specialization of DOM-bacteria networks. **a** We plotted specialization *H*_2_′ against nutrient enrichment for negative (left panel) and positive (right panel) bipartite networks for each elevation in China (red lines) and Norway (blue lines). Statistical significance of linear model fits with one-sided F-statistics was indicated by solid (*P* ≤ 0.05) or dotted (*P* > 0.05) lines. For better visualization, we omitted the data points but detailed scatter plots and statistics are shown in Fig. S14. **b** We examined the relative importance of all explanatory variables on the *H*_2_′ of negative (left panel) and positive (right panel) bipartite networks in China (red lines) and Norway (blue lines) using random forest. The relative contribution (%) of each variable towards *H*_2_′ is shown in radar plots. The explanatory variables were grouped by environment, energy, diversity and traits with consistent colours of ovals or rectangles as in Fig. 6a. Abbreviations of explanatory variables are detailed in Table S1.

Finally, we calculated the degree of specialization between DOM and bacteria in the entire negative and positive interaction networks using the *H*_2_′ index^28^. We also calculated specialization *d*′ indices for individual DOM molecules and bacterial genera^28^. Elevated *H*_2_′ or *d*′ values indicate a high degree of specialization, while lower values suggest increased generalization. That is, highly specialized DOM or bacteria, in the extreme example, mean that a single DOM molecule is used by one bacterial taxon, whereas highly generalized DOM or bacteria mean that different DOM molecules can be used by a large number of bacterial taxa (Fig. 1a). We found that as the negative networks between DOM and bacteria became more specialized (i.e., higher *H*_2_′ values), they corresponded with more specialized DOM molecules (i.e., higher weighted mean *d*′; Fig. S13). More specialized decomposition processes can therefore reduce the vulnerability of DOM to degradation as they will require highly specialized consumers. For positive networks, *H*_2_′ values showed consistent patterns along nutrient enrichment gradients with those of *d*′ for both DOM and bacteria (Fig. S13). This result indicates that increased specialization in production processes was related to the decreased vulnerability of DOM and the decreased generality of microbes, and thus the potentially decreased DOM production. These results collectively suggest that in addition to the specialization perspective of bacteria or DOM, *H*_2_′ can summarise resource-consumer relationships at an ecosystem-level. In both regions, *H*_2_′ was higher, on average, in negative than positive interaction networks (*t*-test, *t* = 2.11, *P* = 0.04 in China and *t* = 23.57, *P* ≤ 0.001 in Norway; Figs. 5a, S14), indicating greater specialization in the decomposition than production processes of microbes. The mean specialization *H*_2_′ of negative (*t*-test, *t* = −10.19, *P* ≤ 0.001) and positive (*t*-test, *t* = −6.56, *P* ≤ 0.001) networks were also significantly higher in Norway than in China (Fig. 5a), suggesting more specialized decomposition (i.e., negative networks) and thus potentially lower vulnerability of DOM decomposition to bacteria in subarctic regions.

Nutrient enrichment showed divergent effects on the *H*_2_′ of negative or positive interaction networks between the two study regions. Specifically, nutrient enrichment substantially decreased the *H*_2_′ of negative networks for all molecules in China (Fig. 5a), which was particularly true when considering only recalcitrant components, such as lignin and CHNO (Fig. S15). Compared to Norway, nutrient enrichment increased the *H*_2_′ of positive interactions relatively more at lower elevations in China (Fig. 5a). Nutrient enrichment in the subtropical region could thus contribute to the greater recalcitrance of DOM by increased decomposition (i.e., less specialized negative networks) and reduced production of molecules (i.e., more specialized positive networks).

### Drivers of DOM-bacteria interaction networks

We explored the effects of distal and proximal drivers on negative and positive DOM-bacteria networks using the EDTiA framework (Fig. 1). The distal drivers were temperature and nutrient enrichment as proxies of climate change and human impacts, respectively. The three proximal drivers were energy supply, such as primary productivity and sediment total organic carbon, the diversity of bacteria and DOM, that is the richness and composition of bacteria and DOM, and DOM molecular traits (Table S1).

We found that, in addition to bacterial diversity and chemodiversity, molecular traits also influenced the network specialization *H*_2_′. In the negative networks, *H*_2_′ was most strongly correlated with DOM molecular composition (*r* = 0.77, *P* ≤ 0.001), followed by molecular richness (*r* = −0.76, *P* ≤ 0.001) and molecular N/P ratio (*r* = 0.76, *P* ≤ 0.001, Fig. S16). In contrast, in the positive networks, *H*_2_′ was less correlated with molecular traits (Fig. S16). Furthermore, we examined the relative importance of diversity and molecular traits for predicting *H*_2_′. *H*_2_′ was mainly affected by chemodiversity, such as molecular richness or DOM composition, followed by molecular traits, such as N/P or N/C ratios, in the negative networks, whereas chemodiversity, biodiversity, environmental variables and energy supply were all similarly important in the positive networks (Fig. 5b).

**Fig. 6:**
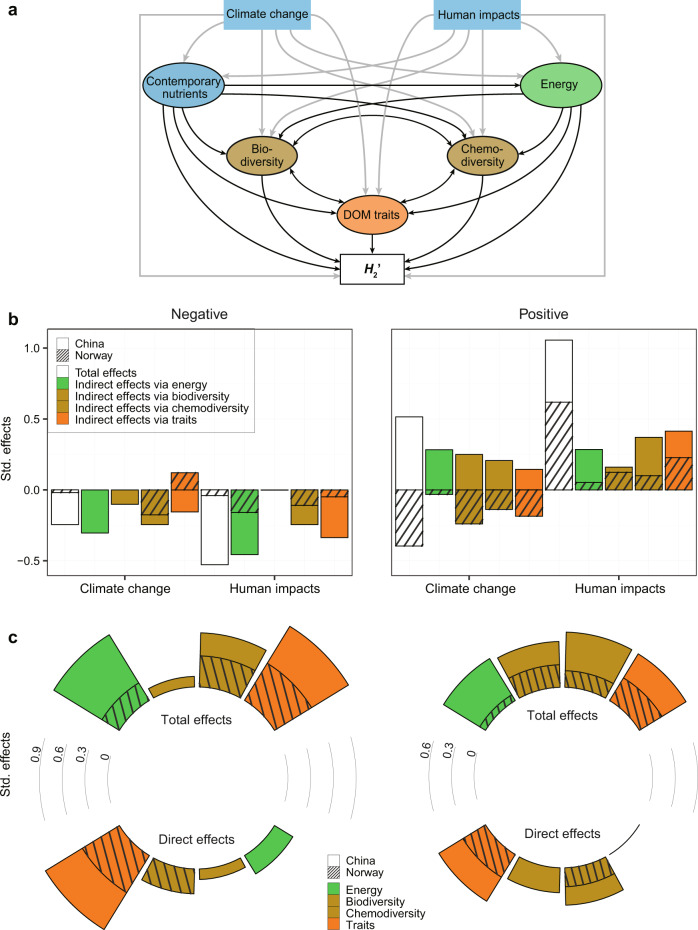
Structural equation models to explain specialization of DOM-bacteria networks. **a** Conceptual model showing the hypothesized causal relationships among distal and proximal drivers for the specialization *H*_2_′ of DOM-bacteria interaction networks. The distal drivers include climate change, human impacts and contemporary nutrients, and the proximal drivers are energy supply, biodiversity, chemodiversity and molecular traits, which are described in detail in Table S3. Grey or black arrows indicate the hypothesized relationships among the exogenous or endogenous composite variables and *H*_2_′, respectively. **b**, **c** Stacked bar plots show the standardised effects (Std. effects) of predictor variables on the *H*_2_′ of negative (left panel) and positive (right panel) bipartite networks in China or Norway estimated from the best supported models. We considered **b** the total and indirect effects of global change and human impacts via proximal variables and **c** the total and direct effects of proximal variables. Details of the full structural equation models are shown in Fig. S17.

We finally tested the hypothesised effects of two global change drivers, temperature change and nutrient enrichment, on the specialization of DOM-bacteria interaction networks. We compared these effects to other drivers such as contemporary nutrients, energy supply, biodiversity, chemodiversity and molecular traits using structural equation models (SEM)^36^ (Fig. 6a). SEM tests hypothesized cause-and-effect relationships, which are translated into regression equations and fitted to data. Through this process, SEM separates the direct and indirect effects of different drivers of *H*_2_′. The SEM results indicated that there were different constraints on the specialization between negative and positive interactions. For the negative interactions, both global change drivers strongly influenced *H*_2_′ through indirect effects on energy supply and molecular traits, especially in China (Figs. 6b, c, S17). In contrast to Norway, both climate change and human impacts had larger total mean effects of −0.23 and −0.49, respectively, on the *H*_2_′ of negative interactions in China (Fig. 6a). However, molecular traits had the dominant direct effects on *H*_2_′ in both China and Norway, with similar mean standardised effect size of 0.57 (*P* ≤ 0.001; Figs. 6b, S17). For the positive interactions, there were large total mean effects of climate change (0.51 and −0.40 for China and Norway, respectively) and human impacts (0.44 and 0.62, respectively), both of which indirectly influenced *H*_2_′ similarly through biodiversity, chemodiversity and molecular traits (Figs. 6b, c, S17).

### Prediction of DOM-bacteria interactions

**Fig. 7:**
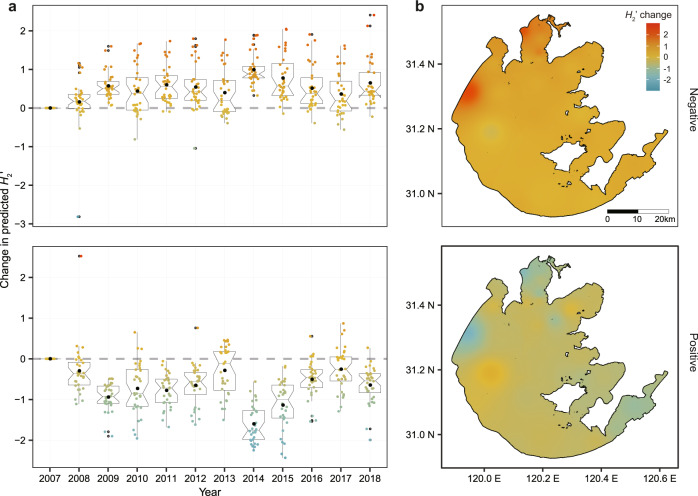
Decadal change in predicted specialization of DOM-bacteria networks in Taihu Lake. **a** Changes in *H*_2_′ of negative (upper panel) and positive (lower panel) bipartite networks from 2007 to 2018. **b** The spatial distribution of changes in *H*_2_′ of negative (upper panel) and positive (lower panel) networks in 2018 across the Taihu Lake. Estimated changes in *H*_2_′ were calculated (*n* = 32 sampling sites across the whole of Taihu Lake; Fig. S19a) by comparing with the baseline of 2007, and represent the combined effects of climate change and eutrophication. The colored dots in **a** indicate *H*_2_′ changes for individual sites which are consistent with the figure legend of (**b**), and black dots are the mean values for each year. The box in **a** represents the interquartile (50% of data), the horizontal line in the box represents the median, the “notch” represents the 95% confidence interval of the median and the “whiskers” represent the maximum and minimum values.

As inland waters worldwide are affected by climate change^37^ and undergo changes in trophic status^38^, our approaches could be used to make predictions about how microbes degrade and produce DOM. For instance, total nitrogen in Taihu Lake has been reduced by a mean (±SD) of 1.24 (±1.41) mg L^−1^ via lake management efforts since a hyper-eutrophication event occurred in May 2007 (Fig. S18). Based on the estimated direct and indirect effects of distal drivers in the SEM fitted to the Chinese data (Fig. 6a), this oligotrophication, combined with a mean decrease in water temperature of 0.20 (±0.87) °C between 2007 and 2018, was predicted to shift DOM-bacteria interaction networks towards more specialized DOM decomposition and more generalized DOM production (Fig. 7). This result indicates that lake management has potentially increased carbon stocks in the lake. Specifically, *H*_2_′ changed by +0.65 (±0.58) and −0.65 (±0.46) for negative and positive interactions, respectively, over this period (Fig. 7a). The greatest changes happened in the most eutrophic part of the lake, including the northwestern lakeshore and the northern Zhushan and Meiliang Bays (Figs. 7b, S19). Although our predictions lack detailed spatiotemporal environmental variation, as used to parameterize the SEM models, they do illustrate the potential to upscale our findings in real-world settings. Ground-truthing our results with in situ measurements across environmental gradients and across spatiotemporal scales could further validate our predictions. Our predictions also highlight how lake management and policy can affect the balance between decomposition and production of organic matter and lake carbon cycling, more generally. Future studies with more lakes or other aquatic systems are needed for a comprehensive understanding of how global change will shift DOM-bacteria interaction networks in inland waters worldwide.

### Implications

The factors that control microbial processing of DOM composition, and consequently its degradation, are complex and challenging to discern^39^, yet are critical for predicting carbon cycling under global change scenarios. We found that associations between DOM and microbial decomposers were influenced by fundamental drivers of ecosystem functioning, such as energy supply^19,20^, both DOM and microbial compositions^9,14^ and molecular traits^8^. The EDTiA framework we developed provides a unified approach from a molecular-level perspective to identify when each of these proximal drivers is more important, and to separate contrasting biological processes associated with DOM degradation and production that may have obscured previous analyses of bulk DOM pools. In addition to energy supply and the diversity of DOM and bacteria, our study reveals that molecular traits are informative for describing DOM-bacteria interaction networks across contrasting climatic zones, especially the negative interactions indicative of degradation processes. Although molecular traits are well known to be linked with DOM persistence or vulnerability to degradation^8,40^, their influence on the underlying biological mechanisms has remained poorly understood. Our results advance this work by demonstrating when the specialization of DOM-microbe networks changes with molecular traits, and by providing predictions of how specialization might vary under global change scenarios.

We found that temperature and nutrient enrichment can change DOM-bacteria interactions by shifting the three proximal drivers, namely energy, diversity, and traits. For the positive bipartite networks, nutrient enrichment generally increased the DOM-bacteria specialization, and more so than temperature, by changing biodiversity, chemodiversity, and molecular traits. Positive interactions related to the production of new molecules depend on the specific interacting partners, which is partly supported by the positive relationships between *H*_2_′ and the *d*′ of DOM or bacteria (Fig. S13). In the negative networks, however, both temperature and nutrient enrichment reduced specialization, primarily via changing molecular traits and energy supply. The greater importance of molecular traits indicates that decomposition processes associated with negative networks may depend more on whether molecules contain structures that resist degradation^8^, especially in the presence of temperature and nutrient limitation^41^. At lower temperatures and nutrient levels, the required activation energy to degrade these molecules may become more limiting^41^. We also found that the importance of these distal drivers of climate change and human impacts varied between biomes. For instance, both elevated temperature and nutrient enrichment reduced the specialization of negative DOM-bacteria interactions in subtropical China, but these two drivers were less important in subarctic Norway. As their indirect effects via microbial composition varied between biomes, these responses may partly reflect differences in the functional traits and activity of biological communities. Such mechanisms via the three proximal drivers may also help evaluate how the biological approaches such as biochemical oxygen demand could be applied in assessing dissolved organic carbon dynamics in natural waters^42^. Future studies with metagenomics and metatranscriptomics could offer a powerful complement to test how microbial traits vary with DOM traits.

In summary, we found that DOM-microbe associations were primarily influenced by temperature change and nutrient enrichment via energy, diversity and traits, the integration of which is a requisite for predicting how organic carbon responds to multiple global change drivers. Looking forward, there is a need to translate the DOM-microbe associations of our EDTiA framework into process-based ecosystem models, from which predictions of the future carbon cycle stand to be improved by incorporating more information on microbial community function, such as their specialization on different DOM sources^43,44^. More generally, our work shows how the molecular traits of DOM control the responses of DOM-microbe networks and their associated biogeochemical cycles in a changing world.

## Methods

### Experimental design

The comparative field microcosm experiments were conducted on Laojun Mountain in China (26.6959 N; 99.7759 E) in September–October 2013, and on Balggesvarri Mountain in Norway (69.3809 N; 20.3483 E) in July 2013, designed to be broadly representative of subtropical and subarctic climatic zones, respectively, as first reported in Wang et al.^29^. In the Laojun Mountain region, mean annual temperatures ranged from 4.2 to 12.9 °C, with July mean temperatures of 17–25 °C. In the Balggesvarri Mountain region, mean annual temperatures ranged from −2.9 to 0.7 °C, with July mean temperatures of 8–16 °C. The experiments were characterised by an aquatic ecosystem with consistent initial DOM composition but different locally colonised microbial communities and newly produced endogenous DOM. While allowing us to minimise the complexity of natural ecosystems, the experiment provided a means for investigating DOM-microbe associations at large spatial scales by controlling the initial DOM supply. Briefly, we selected locations with five different elevations on each mountainside. The elevations were 3822, 3505, 2915, 2580 and 2286 m a.s.l. on Laojun Mountain in China, and 750, 550, 350, 170 and 20 m a.s.l. on Balggesvarri Mountain in Norway. At each elevation, we established 30 aquatic microcosms (1.5 L bottle) composed of 15 g of sterilised lake sediment and 1.2 L of sterilised artificial lake water at one of ten nutrient levels of 0, 0.45, 1.80, 4.05, 7.65, 11.25, 15.75, 21.60, 28.80 and 36.00 mg N L^−1^ of KNO_3_ in the overlying water. To compensate for nitrate additions shifting stoichiometric ratios, KH_2_PO_4_ was added to the bottles so that the N/P ratio of the initial overlying water was 14.93, which was similar to the annual average ratio in Taihu Lake during 2007 (that is, 14.49). Thus, we use “nutrient enrichment” to indicate a series of targeted nutrient levels of both nitrate and phosphate, the former of which was used to represent nutrient enrichment in the statistical analyses. Each nutrient level was replicated three times. The lake sediments were obtained from the centre of Taihu Lake, China, and were aseptically canned per bottle after autoclaving at 121 °C for 30 min. Nutrient levels for the experiments were selected based on conditions of the eutrophic Taihu Lake, and the highest nitrate concentration was based on the maximum total nitrogen in 2007 (20.79 mg L^−1^; Fig. S19). We chose the nutrient level of this year because a massive cyanobacteria bloom in Taihu Lake happened in May 2007 and initiated an odorous drinking water crisis in the nearby city of Wuxi.

The microcosms were left in the field for one month allowing airborne bacteria to freely colonise the sediments and water. To keep the microbial dispersal events as natural as possible, we did not cover the experimental microcosms in case of rainfall. To avoid or minimize potential influence of extreme nature events, we (i) left the top 20% of each microcosm empty to prevent water from overflowing during heavy rains, and (ii) checked the experimental sites twice during each experimental period, and added sterilized water to obtain a final volume of approximately 1.2 L. The bottom of our microcosm was buried into the local soils by 10% of the bottle height, partly to reduce UV exposure to sediments. More considerations of the experimental design were detailed in the Supplementary Methods. To avoid the effects of daily temperature variation, we measured the water temperature and pH within 2 h before noon at all elevations in the day before the final sample collection. At the end of the experimental period, we aseptically sampled the water and sediments of the 300 bottles (that is, 2 mountains × 5 elevations × 10 nutrient levels × 3 replicates) for the following analyses of physiochemical variables, bacterial community and DOM composition.

### Physiochemical variables and bacterial community

We measured environmental variables, namely, the total nitrogen (TN), total phosphorus (TP), dissolved nutrients (that is, NO_x_^−^, NO_2_^−^, NH_4_^+^ and PO_4_^3−^), total organic carbon (TOC), dissolved organic carbon (DOC) and chlorophyll *a* (Chl *a*) in the sediments, and the NO_3_^−^, NO_2_^−^, NH_4_^+^, PO_4_^3−^ and pH in the overlying water (Table S2, Fig. S20), according to Wang et al.^29^.

The sediment bacteria were examined using high-throughput sequencing of 16S rRNA genes. The sequences were processed in QIIME (v1.9)^45^ and OTUs were defined at 97% sequence similarity. The bacterial sequences were rarefied to 20,000 per sample. Further details on physicochemical and bacterial community analyses are available in Wang et al.^29^.

### ESI FT-ICR MS analysis of DOM samples

Highly accurate mass measurements of DOM within the sediment samples were conducted using a 15 Tesla solariX XR system, a ultrahigh-resolution Fourier transform ion cyclotron resonance mass spectrometer (FT-ICR MS, Bruker Daltonics, Billerica, MA) coupled with an electrospray ionization (ESI) interface, as demonstrated previously^46^ with some modifications. It should be noted that FT-ICR MS does not identify molecules, but only molecular formulae in terms of elemental composition and there can be many molecular structures sharing the same elemental compositions. DOM was solid-phase extracted (SPE) with Agilent VacElut resins before FT-ICR MS measurement^47^ with minor modifications. Briefly, an aliquot of 0.7 g freeze-dried sediment was sonicated with 30 ml ultrapure water for 2 h, and centrifuged at 5000 × *g* for 20 min. The extracted water was filtered through the 0.45 μm Millipore filter and further acidified to pH 2 using 1 M HCl. Cartridges were drained, rinsed with ultrapure water and methanol (ULC-MS grade), and conditioned with pH 2 ultrapure water. Calculated volumes of extracts were slowly passed through cartridges based on DOC concentration. Cartridges were rinsed with pH 2 ultrapure water and dried with N_2_ gas. Samples were finally eluted with methanol into precombusted amber glass vials, dried with N_2_ gas and stored at −20 °C until DOM analysis. The extracts were continuously injected into the standard ESI source with a flow rate of 2 μl min^−1^ and an ESI capillary voltage of 3.5 kV in negative ion mode. One hundred single scans with a transient size of 4 mega word (MW) data points, an ion accumulation time of 0.3 s, and within the mass range of *m*/*z* 150–1200, were co-added to a spectrum with absorption mode for phase correction, thereby resulting in a resolving power of 750,000 (FWHM at *m*/*z* 400). All FT-ICR mass spectra were internally calibrated using organic matter homologous series separated by 14 Da (-CH_2_ groups). The mass measurement accuracy was typically within 1 ppm for singly charged ions across a broad *m*/*z* range (150–1200 *m*/*z*).

Data Analysis software (BrukerDaltonik v4.2) was used to convert raw spectra to a list of *m*/*z* values using FT-MS peak picker with a signal-to-noise ratio (S/N) threshold set to 7 and absolute intensity threshold to the default value of 100. Putative chemical formulae were assigned using the software Formularity (v1.0)^48^ following the Compound Identification Algorithm^49^. In total, 19,538 molecular formulas were putatively assigned for all samples (*n* = 300) based on the following criteria: S/N > 7, and mass measurement error <1 ppm, considering the presence of C, H, O, N, S and P and excluding other elements or an isotopic signature. All formula assignments were further screened to meet the criteria as follows^50^: (1) formulae containing an odd number of nitrogen atoms had an even nominal m/z and those containing an even number of nitrogen atoms had an odd nominal *m*/*z*; (2) the number of hydrogen atoms was at least 1/3 of carbon and could not exceed 2C + N + 2; (3) the number of nitrogen or oxygen atoms could not exceed the number of carbon atoms; (4) the ratio of O/C was set to 0–1, H/C ≥ 0.3, N/C ≤ 1, double bond equivalents (DBE) ≥ 0.

The assigned molecules were categorised into eight compound classes or 12 elemental combinations. The compound classes based on van Krevelen diagrams^51^ were lipids (O/C = 0–0.3, H/C = 1.5–2.0), proteins (O/C = 0.3–0.55, H/C = 1.5–2.2), amino sugars (O/C = 0.55–0.67, H/C = 1.5–2.2), carbohydrates (Carb; O/C = 0.67–1.2, H/C = 1.5–2), unsaturated hydrocarbons (UnsatHC; O/C = 0-0.1, H/C = 0.7–1.5), lignin (O/C = 0.1–0.67, H/C = 0.7–1.5), tannin (O/C = 0.67–1.2, H/C = 0.5–1.5) and condensed aromatics (ConHC; O/C = 0–0.67, H/C = 0.2–0.7). The elemental combinations were CH, CHN, CHNO, CHNOP, CHNOS, CHNOSP, CHNS, CHO, CHOP, CHOS, CHOSP and CHS.

### Estimating DOM features

We considered DOM features from three aspects: alpha diversity, beta diversity and molecular traits. These features were considered for all molecules (19,538 different formulae), but also for subsets of molecules within each category of compound classes or elemental combinations. The dataset based on all molecular formulae was named “All molecules”, while the datasets of subsets of formulae were named by “category name + compounds”. The relative abundance of molecules was calculated by normalizing signal intensities of assigned peaks to the sum of all intensities within each sample. Peak (i.e., molecule) intensity may not always necessarily provide reliable information on absolute concentrations. Therefore, we did not analyse absolute intensities but instead standardised the intensity relative to all other molecules in a sample. The measure of relative abundance has been shown to be very informative in revealing ecological patterns in previous studies, such as along soil depth^9^ and in lakes^8^, and so should help determine how temperature and nutrient enrichment influence DOM-microbe associations. Notably, there are also methodological reports supporting the quantitative aspect of high-resolution mass spectrometry method. For instance, the intensity of compounds varies linearly with their initial concentration, and this linear relationship was quite similar among compounds, especially when coupled with charged aerosol detection^52^. More importantly, our experimental design used a common pool of homogenized sediments across experimental units such that the overall matrix of organic molecules may be assumed to be relatively similar across samples. This provides a greater confidence that a change in peak intensity for a given molecule reflects a relative change in its concentration across microcosms (i.e., samples) and the two main environmental gradients.

We considered two aspects of chemodiversity: chemical alpha diversity and chemical beta diversity. Chemical alpha diversity was calculated using a richness index that counts the total number of peaks in each sample. Chemical beta diversity was calculated with the Bray-Curtis dissimilarity metric, and further represented by the first two axes of a non-metric multidimensional scaling (NMDS) ordination of this dissimilarity. We also considered overall molecular composition, which was visualised across the elevations and nutrient enrichment treatments with detrended correspondence analysis (DCA)^53^. The analyses of chemical diversity were performed using the R package vegan V2.4.6^54^.

We also calculated 16 molecular traits that could affect microbial associations and were related to molecular weight, stoichiometry, chemical structure, and oxidation state (Table S1). These traits were mass, the number of carbon (C) atoms, the modified aromaticity index (AI_Mod_)^55^, DBE^55^, DBE minus oxygen (DBE_O_)^55^, DBE minus AI (DBE_AI_)^55^, standard Gibb’s Free Energy of carbon oxidation (GFE)^56^, Kendrick Defect (kdefect_CH2_)^57^, nominal oxidation state of carbon (NOSC), O/C ratio, H/C ratio, N/C ratio, P/C ratio, S/C ratio, and carbon use efficiency (Y_met_)^58^. All calculations were performed using the R package ftmsRanalysis V1.0.0^59^ and the scripts at https://github.com/danczakre/ICRTutorial. DBE represents the number of unsaturated bonds and rings in a molecule^55^. Higher values of DBE, AI and NOSC all indicate a higher recalcitrance of DOM. A large Kendrick Defect can indicate a higher degree of oxidation. Lower values of Y_met_ indicate a higher thermodynamic efficiency of metabolic reactions involved in biomass production^58^. Weighted means of formula-based molecular traits (for example the Mass_wm_ for Mass) were calculated as the sum of the product of the trait value for each individual molecule (Mass_*i*_) and relative intensity *I*_*i*_ divided by the sum of all intensities with the R package FD V1.0.12^60^ using the equation:

1 $${{{{{{\rm{Mass}}}}}}}_{{{{{{\rm{wm}}}}}}}=\frac{\sum {{{{{{\rm{Mass}}}}}}}_{i}\times {I}_{i}}{\sum ({I}_{i})}$$

In addition, ten clusters of molecules were grouped based on the 16 molecular traits by hierarchical cluster analysis using Ward’s minimum variance method with the R package stats V3.6.1.

### Characterising bacterial communities

The relative abundance of OTUs was calculated by the normalization of read counts of OTUs to the sum of all reads within each sample. Likewise, we considered two aspects of biodiversity: bacterial alpha diversity and beta diversity. Bacterial alpha diversity was calculated using species richness that counts the total number of OTUs in each sample. Bacterial beta diversity was calculated with the Bray-Curtis dissimilarity metric, and further represented by the first two axes of NMDS of this dissimilarity.

### Estimating associations between DOM and bacteria

At the DOM compositional level, we examined DOM-bacteria associations from the following aspects: Pearson’s correlation between alpha diversity of DOM and bacteria, and a Mantel correlation between the beta diversity of DOM and bacteria (Box 1). We also tested the congruence between DOM and bacterial composition using Procrustes analysis of NMDS coordinates estimated for each community across elevations and nutrient enrichment levels with the Bray-Curtis dissimilarity metric^32,33^. Procrustes analysis is a technique for comparing the relative positions of points (i.e., samples or sites) in two multivariate datasets (in an ordination space). It attempts to stretch and rotate the points in one matrix, such as points obtained from a NMDS, to be as close as possible to points in another matrix, thus preserving the relative distances between points within each matrix^32,33^. This procedure yields a measure of fit, *M*^2^, which is the sum of squared distances between corresponding data points after the transformation. Analogous to a Mantel test, Procrustes analysis is particularly used to determine how much variance in one matrix (i.e., bacteria) is attributable to the variance in the other (i.e., DOM) or to assess the statistical significance in the correlation between the two multivariate datasets. In addition, Procrustes analysis has the advantages of the application of the Procrustean association metric (i.e., residuals). Pointwise residuals indicate the difference between two different community ordinations for each sample, and are used to examine how the DOM-bacteria associations could be influenced by environmental gradients such as elevations and nutrients. The statistical significance of the Procrustes analysis (i.e., *M*^2^) can then be assessed by randomly permutating the data 1000 times^61^. This analysis was performed using the R package vegan V2.4.6.

We further quantified DOM-bacteria associations at a molecular level using two different co-occurrence analyses (Box 1). First, Spearman’s rank correlation coefficient *ρ* was calculated between the relative abundance of each molecule *m*/*z* ion and bacterial OTU (or genus). For each molecule, we then calculated the Spearman *ρ* difference by subtracting the mean absolute *ρ* value of the negative correlations across all bacterial OTUs from the mean of the positive correlations. Larger positive and negative values indicate that molecules were more strongly positively and negatively correlated with bacterial communities, respectively. The relationships among the Spearman *ρ* difference, H/C and O/C were summarised using kriging interpolation with the R package automap V1.0.14^62^. Second, SparCC (Sparse Correlations for Compositional data) was applied to build DOM-bacteria bipartite networks. SparCC is a correlation method that can infer the interrelationships between DOM and bacteria for compositional data with higher accuracy^35^ than general correlation approaches, such as Spearman’s correlation, because it explicitly assumes that the underlying networks have many missing associations. We used bacterial genera rather than OTUs for bipartite network analysis because there were over 20,000 and 10,000 bacterial OTUs for Norway and China, respectively, and there are computational limits on handling such large bipartite networks for the analyses described in the next paragraph. However, using bacterial genera was reasonable as individual DOM-bacteria interactions were similar for both bacterial OTUs and genera (*R*^2^ > 0.80, *P* ≤ 0.001; Fig. S9). Similar conclusions were also obtained with either OTUs or genera when relating the pairwise distances of molecular traits with SparCC correlation coefficient *ρ* values among DOM molecules in Fig. 4c. To reduce type I errors in the correlation calculations created by low-occurrence genera or molecules, the majority rule was applied; that is, we retained genera or molecules that were observed in more than half of the total samples (≥75 samples) in China or Norway. The filtered table, including 1340 and 1246 DOM molecules, and 75 and 49 bacterial genera in China and Norway, respectively, was then used for pairwise correlation calculation of DOM and bacteria using SparCC with default parameters^35^.

Finally, bipartite network analysis at a molecular level was performed to quantify the specialization of DOM-bacteria networks (Box 1). The specialization considers interaction abundance and is standardised to account for heterogeneity in the interaction strength and species richness, which describes the levels of “vulnerability” of DOM molecules and “generality” of bacterial taxa^27^. The threshold correlation for inclusion in bipartite networks was |*ρ*| = 0.30 to exclude weak interactions and we retained the adjacent matrix with only the interactions between DOM and bacteria. We then constructed two types of interaction networks (i.e., negative and positive networks) based on negative and positive correlation coefficients (SparCC *ρ* ≤ −0.30 and *ρ* ≥ 0.30, respectively). According to resource-consumer relationships, negative networks likely indicate the degradation of larger molecules into smaller structures, while positive networks may suggest the production of new molecules via degradation or biosynthetic processes. The SparCC *ρ* values were multiplied by 10,000 and rounded to integers, and the absolute values were taken for negative networks to enable the calculations of specialization indices. A separate negative and positive sub-network was obtained for each microcosm by selecting the DOM molecules and bacterial taxa in each sample based on its bacterial and DOM compositions. For the network level analysis, we calculated *H*_2_′, a measure of specialization^27^, for each network:

2 $${H}_{2}=-\mathop{\sum }\limits_{i{{\mbox{=}}}1}^{i}\mathop{\sum }\limits_{j{{\mbox{=}}}1}^{j}{{\mbox{(}}}{{{\mbox{p}}}}_{{ij}}{{{{{{\rm{ln}}}}}}}{{{\mbox{p}}}}_{{ij}}{{\mbox{)}}}$$

3 $${H}_{2}{\prime} =\frac{{H}_{2{\max }}{-}{H}_{2}}{{H}_{2{\max }}{-}{H}_{2{\min }}}$$

where ${{{\mbox{p}}}}_{{ij}}{{\mbox{=}}}{{{\mbox{a}}}}_{{ij}}{{\mbox{/}}}m$, represents the proportion of interactions in a *i* × *j* matrix. ${{{\mbox{a}}}}_{{ij}}$ is the number of interactions between DOM molecule *i* and bacterial genus *j*, which is also referred as “link weight”. *m* is the total number of interactions between all DOM molecules and bacterial genera. *H*_2_′ is the standardised *H*_2_ against the minimum (*H*_2min_) and maximum (*H*_2max_) possible for the same distribution of interaction totals. For the molecular level analysis, we calculated the specialization index Kullback–Leibler distance (*d*′) for DOM molecules (*d*_*i*_′) and bacterial genera (*d*_*j*_′), which describes the levels of “vulnerability” of DOM molecules and “generality” of bacterial genera, respectively:

4 $${d}_{i}=\mathop{\sum }\limits_{j=1}^{j}\left(\frac{{{{\mbox{a}}}}_{{ij}}}{{{{\mbox{A}}}}_{i}}{{{\mbox{ln}}}}\frac{{{{\mbox{a}}}}_{{ij}}m}{{{{\mbox{A}}}}_{i}{{{\mbox{A}}}}_{j}}\right)$$

5 $${d}_{i}{\prime} =\frac{{d}_{i}-{d}_{{\min }}}{{d}_{{\max }}-{d}_{{\min }}}$$

where ${A}_{i}$ = $\mathop{\sum }\limits_{j{{\mbox{=}}}1}^{j}{{{\mbox{a}}}}_{{ij}}$ and ${A}_{j}$ = $\mathop{\sum }\limits_{i{{\mbox{=}}}1}^{i}{{{\mbox{a}}}}_{{ij}}$, are the total number of interactions of DOM molecule *i* and bacterial genus *j*, respectively. *d*_*i*_′ is the standardised *d*_*i*_ against the minimum (*d*_min_) and maximum (*d*_max_) possible for the same distribution of interaction totals. The equations of *d*_*j*_′ are analogous to *d*_*i*_′, replacing *j* by *i*. Weighted means of *d*′ for DOM were calculated for each network as the sum of the product of *d*′ for each individual molecule *i* (*d*_*i*_′) and relative intensity *I*_*i*_ divided by the sum of all intensities *d*′  = Ʃ(*d*_*i*_′ × *I*_*i*_)/Ʃ(*I*_*i*_). Weighted means of *d*′ for bacteria were calculated as the sum of the *d*′ of each individual bacterial genus *j* (*d*_*j*_′) and relative abundance of bacterial genus *I*_*j*_ divided by the sum of all abundance. All calculations were performed using the R package FD V1.0.12. The observed *H*_2_′ and *d*′ values ranged from 0 (complete generalization) to 1 (complete specialization)^28^ (Fig. S21). Specifically, elevated *H*_2_′ or *d*′ values indicate a high degree of specialization, while lower values suggest increased generalization, that is, higher vulnerability of DOM and/or higher generality of microbes. To directly compare the network indices across the elevations or nutrient enrichment levels, we used a null modelling approach. We standardised the three observed specialization indices (*S*_observed_; that is, *H*_2_′, *d*′ of DOM_,_ and *d*′ of bacteria) by calculating their z-scores^63^ using the equation:

6 $${z}_{S}=({S}_{{{{{{\rm{observed}}}}}}}-\overline{{{S}}_{{{{{{\rm{null}}}}}}}})/({\sigma }_{S_{{{{{\rm{null}}}}}}})$$

where $\overline{{{S}}_{{{{{{\rm{null}}}}}}}}$ and ${\sigma }_{S_{{{{{\rm{null}}}}}}}$ were, respectively, the mean and standard deviation of the null distribution of *S* (*S*_null_). One hundred randomised null networks were generated for each bipartite network to derive *S*_null_ using the *swap.web* algorithm, which keeps species richness and the number of interactions per species constant along with network connectance. This null model analysis indicates that interactions between DOM and bacteria were non-random as the observed network specialization indices were generally significantly lower than expected by chance (*P* < 0.05). The obtained network was visualised using circlize V0.4.10^64^ and analysed using the R package bipartite V2.15^27^.

### Statistical analyses

We analysed how DOM features and DOM-bacteria associations varied with temperature and nutrient enrichment at both compositional- and molecular-levels, which were outlined in Fig. 2. We used the following abiotic and biotic drivers explaining variations in DOM features (i.e., alpha diversity, beta diversity, and molecular traits): water temperature, nutrient enrichment (i.e., the experimental addition of nitrate and phosphate), contemporary nutrients (i.e., TN, TP, NO_x_^−^, NO_2_^−^, NH_4_^+^ and PO_4_^3−^ in the sediments, and NO_3_^−^, NO_2_^−^, NH_4_^+^ and PO_4_^3−^ in the overlying water), energy supply (i.e., sediment TOC, DOC, water pH and sediment Chl *a*), and biodiversity (i.e., the species richness and the first two axes of the NMDS of bacterial community composition) (Table S2). In addition, for the response variables of DOM-bacteria associations, we used the following explanatory variables related to distal and proximal drivers (Table S1). Distal environmental drivers included climate change (i.e., water temperature), human impacts (i.e., nutrient enrichment), and contemporary nutrients (i.e., TN, TP, NO_x_^−^, NO_2_^−^, NH_4_^+^ and PO_4_^3−^, and water NO_3_^−^, NO_2_^−^, NH_4_^+^ and PO_4_^3−^). Proximal drivers included energy supply (i.e., sediment TOC, DOC, water pH and sediment Chl *a*), biodiversity (i.e., the species richness and the first two axes of the NMDS of bacterial community composition), DOM chemodiversity (i.e., the species richness and the first two axes of the NMDS of molecular composition), and DOM molecular traits (i.e., mass, C, AI_Mod_, DBE, DBE_O_, DBE_AI_, GFE, kdefect_CH2_, NOSC, O/C, H/C, N/C, P/C, S/C and Y_met_). It should be noted that water pH could be considered to be relevant to primary productivity due to its strong positive correlation with sediment Chl *a*, but their relationships varied across elevations and nutrient levels^29^.

For DOM features, the relationships between nutrient enrichment and DOM richness or molecular traits were visualised with linear models for all formulae and subsets of formulae within each category of compound classes or elemental combinations across different elevations. We further tested the breakpoints or abrupt changes in DOM composition (i.e., the first axis of DCA) along the gradient of nutrient enrichment using a piecewise linear regression with the R package segmented V1.3.0^31^. The number of breakpoints was selected with the following criteria: the Bayesian Information Criteria (BIC) statistics for model selection and a maximum number of three breakpoints. We did not specify the initial nutrient values for breakpoint so that any values ranging from 0 to 36 mg N L^−1^ would be considered. These breakpoint estimations were further supported by gradient forest analysis^30^, which was used to assess the DOM compositional changes and important breakpoints across multiple molecules along the gradient of nutrient enrichment. Gradient forest analysis aggregates random forest models estimated for each molecule along the nutrient gradient^30^, allowing us to identify non-linear changes in overall composition. This analysis produces the standardised density of splits (that is, breakpoints), that is the kernel density of splits divided by the observation density, which shows the distribution of breakpoints of each molecule regarding its abundance occurring along the nutrient gradient^30^. In addition, we estimated the standardised density of splits for subsets of molecules within each category of compound classes or elemental combinations across different elevations. This analysis was performed using the R packages gradientForest V0.1.17^30^ and extendedForest V1.6.1^65^.

For DOM-bacteria associations, the relationships between nutrient enrichment and associations at both community and network levels were tested with linear models for all formulae and subsets of formulae within each category of compound classes or elemental combinations across different elevations.

To further evaluate the key drivers of DOM features and DOM-bacteria associations, we used variation partitioning analysis (VPA)^66^, random forest analysis^67^ and structural equation modelling (SEM)^36^. In particular, the first analysis disentangled the important roles of bacteria from other explanatory variables, while the other non-linear and linear analyses tested the roles of molecular traits and diversity, and their interplay with environments and energy supply.

First, VPA was used to quantify the relative contributions of driver categories towards DOM features. We partitioned explanatory variables into the following driver categories: environments (that is, climate change, human impacts and contemporary nutrients), energy supply and biodiversity (Table S2). We selected explanatory variables for regression analyses by forward selection with Akaike information criterion (AIC)^68^. We also quantified the relative contributions of driver categories for subsets of molecules within each category of compound classes or elemental combinations. VPA was performed with R package vegan V2.4.6^69^.

Second, random forest analysis was conducted to identify the relative importance of environment variables, energy supply, bacterial diversity and DOM molecular drivers on specialization *H*_2_′. The importance of each predictor variable was determined by evaluating the decrease in prediction accuracy (that is, increase in the mean square error between observations and out-of-bag predictions) when the data for that predictor were randomly permuted. The accuracy importance measure was computed for each tree and averaged over the forest (2000 trees). More details on this method were described in previous literature^70^. This analysis was conducted using the R package randomForestSRC V2.8.0^71,72^.

Third, SEM was used to explore how specialization *H*_2_′ is interactively influenced by global changes (that is, temperature and nutrient enrichment), diversity and molecular traits. The approach begins by hypothesising the underlying structure of causal links as shown in Fig. 6a. Then, the model is translated into regression equations, and these equations are evaluated against the data to test the hypothesised links. Through this process, SEM provides an understanding of direct and indirect links of climate change and human impacts on *H*_2_′. Before modelling, all variables in the SEMs were z-score transformed to allow comparisons among multiple predictors and models. Similar to previous studies^73^, we used composite variables to account for the collective effects of climate change, human impacts, contemporary nutrients, energy supply, biodiversity, chemodiversity and molecular traits, and the candidate observed indicators are given in Table S1. The indicators for each composite were selected based on the multiple regressions for *H*_2_′ (Table S3). Based on all the hypothesised links among composite variables (that is, full model; Fig. 6a), we examined all alternative models using AIC and overall model fit statistics^74^. We chose the final model to report as that with the lowest AIC value from models with a non-significant *χ*^2^ test (*P* > 0.05), which tests whether the model structure differs from the observed data, high comparative fit index (CFI > 0.95) and low standardised root mean squared residual (SRMR < 0.05) (Table S4). We implemented the SEMs using R package lavaan V.0.5.23^75^.

### Predictions of DOM-bacteria network specialization in Taihu Lake

Using the parameter estimates obtained from SEM fitted to the bipartite networks in subtropical China, we estimated spatiotemporal variation of DOM-bacteria network specialization in Taihu Lake based on the direct and indirect effects of climate change and eutrophication via the proximal drivers. We first formulated five linear equations to predict the values of contemporary nutrients (*P*_nut_), energy supply (*P*_energy_), biodiversity (*P*_biodiv_), chemodiversity (*P*_chemodiv_) and molecular traits (*P*_trait_) based on climate and eutrophication drivers:

7 $${P}_{{{{{{\rm{nut}}}}}}}={\lambda }_{{{{{{\rm{nut}}}}}},{{{{{\rm{temp}}}}}}}\times {X}_{{{{{{\rm{T}}}}}}}+{\lambda }_{{{{{{\rm{nut}}}}}},{{{{{\rm{N}}}}}}}\times {X}_{{{{{{\rm{N}}}}}}}$$

8 $${P}_{{{{{{\rm{energy}}}}}}}={\lambda }_{{{{{{\rm{energy}}}}}},{{{{{\rm{temp}}}}}}}\times {X}_{{{{{{\rm{T}}}}}}}+{\lambda }_{{{{{{\rm{energy}}}}}},{{{{{\rm{N}}}}}}}\times {X}_{{{{{{\rm{N}}}}}}}+{\lambda }_{{{{{{\rm{energy}}}}}},{{{{{\rm{nut}}}}}}}\times {P}_{{{{{{\rm{nut}}}}}}}$$

9 $${P}_{{{{{{\rm{biodiv}}}}}}}={\lambda }_{{{{{{\rm{biodiv}}}}}},{{{{{\rm{temp}}}}}}}\times {X}_{{{{{{\rm{T}}}}}}}+{\lambda }_{{{{{{\rm{biodiv}}}}}},{{{{{\rm{N}}}}}}}\times {X}_{{{{{{\rm{N}}}}}}}+{\lambda }_{{{{{{\rm{biodiv}}}}}},{{{{{\rm{nut}}}}}}}\times {P}_{{{{{{\rm{nut}}}}}}}+{\lambda }_{{{{{{\rm{biodiv}}}}}},{{{{{\rm{energy}}}}}}}\times {P}_{{{{{{\rm{energy}}}}}}}$$

10 $${P}_{{{{{{\rm{chemodiv}}}}}}}= 	\;{\lambda }_{{{{{{\rm{chemodiv}}}}}},{{{{{\rm{temp}}}}}}}\times {X}_{{{{{{\rm{T}}}}}}}+{\lambda }_{{{{{{\rm{chemodiv}}}}}},{{{{{\rm{N}}}}}}}\times {X}_{{{{{{\rm{N}}}}}}}+{\lambda }_{{{{{{\rm{chemodiv}}}}}},{{{{{\rm{nut}}}}}}}\times {P}_{{{{{{\rm{nut}}}}}}}\\ 	+{\lambda }_{{{{{{\rm{chemodiv}}}}}},{{{{{\rm{energy}}}}}}}\times {P}_{{{{{{\rm{energy}}}}}}}$$

11 $${P}_{{{{{{\rm{trait}}}}}}}= 	\;{\lambda }_{{{{{{\rm{trait}}}}}},{{{{{\rm{temp}}}}}}}\times {X}_{{{{{{\rm{T}}}}}}}+{\lambda }_{{{{{{\rm{trait}}}}}},{{{{{\rm{N}}}}}}}\times {X}_{{{{{{\rm{N}}}}}}}+{\lambda }_{{{{{{\rm{trait}}}}}},{{{{{\rm{nut}}}}}}}\times {P}_{{{{{{\rm{nut}}}}}}}+{\lambda }_{{{{{{\rm{trait}}}}}},{{{{{\rm{energy}}}}}}}\times {P}_{{{{{{\rm{energy}}}}}}}\\ 	+{\lambda }_{{{{{{\rm{trait}}}}}},{{{{{\rm{biodiv}}}}}}}\times {P}_{{{{{{\rm{biodiv}}}}}}}+{\lambda }_{{{{{{\rm{trait}}}}}},{{{{{\rm{chemodiv}}}}}}}\times {P}_{{{{{{\rm{chemodiv}}}}}}}$$

where *X*_T_ and *X*_N_ were water temperature and total nitrogen, respectively, for the 32 sites across the whole Taihu Lake (Fig. S19a). The abbreviations of path coefficients (*λ*) are detailed in Table S5.

Similarly, we calculated the specialization of DOM-bacteria networks (*Y*_H2_) using a linear equation:

12 $${Y}_{{{{{{\rm{H}}}}}}2}= 	\;{\lambda }_{{{{{{\rm{H}}}}}}2,{{{{{\rm{temp}}}}}}}\times {X}_{{{{{{\rm{T}}}}}}}+{\lambda }_{{{{{{\rm{H}}}}}}2,{{{{{\rm{N}}}}}}}\times {X}_{{{{{{\rm{N}}}}}}}+{\lambda }_{{{{{{\rm{H}}}}}}2,{{{{{\rm{nut}}}}}}}\times {P}_{{{{{{\rm{nut}}}}}}}+{\lambda }_{{{{{{\rm{H}}}}}}2,{{{{{\rm{energy}}}}}}}\times {P}_{{{{{{\rm{energy}}}}}}}\\ 	+{\lambda }_{{{{{{\rm{H}}}}}}2,{{{{{\rm{biodiv}}}}}}}\times {P}_{{{{{{\rm{biodiv}}}}}}}+{\lambda }_{{{{{{\rm{H}}}}}}2,{{{{{\rm{chemodiv}}}}}}}\times {P}_{{{{{{\rm{chemodiv}}}}}}}+{\lambda }_{{{{{{\rm{H}}}}}}2,{{{{{\rm{trait}}}}}}}\times {P}_{{{{{{\rm{trait}}}}}}}$$

We used the predicted values for contemporary nutrients, energy supply, biodiversity, chemodiversity and molecular traits in the overall prediction model to account for the indirect effects of water temperature and total nitrogen on specialization. The models were calculated with a yearly time step based on the annual means of water temperature and total nitrogen for each site during 2007–2018. The temporal changes in specialization were calculated using 2007 as a baseline to which all predictions were compared.

The above predictions aimed to apply our EDTiA framework to estimate changes in DOM-bacteria network specialization under temperature change and eutrophication in Taihu Lake, and potential uncertainties in the estimated specialization should however be noted as follows. First, local environmental variation (e.g., N/P ratio changes) and different microbial species pools between our field microcosms and natural lake sediments would likely influence the accuracy of predictions. Second, spatial and temporal heterogeneity of sediments would influence local environments and the composition of both DOM and microbes and thus the projection of estimates across Taihu Lake. Third, the transferability and extrapolation of SEM models to Taihu Lake would be one of the difficulties in prediction practices. We thus selected the SEM models in China rather than Norway for more similar climatic conditions to the target lake. The annual mean water temperatures in Taihu Lake were covered by the temperature variations across the elevations between 2286 and 3822 m a.s.l. in Laojun Mountain, and the annual mean total nitrogen fell into the gradient of nutrient concentrations between 0 and 36 mg N L^−1^. Finally, lake management such as mechanical removal of algae would affect energy supply and consequently prediction accuracy.

### Reporting summary

Further information on research design is available in the Nature Research Reporting Summary linked to this article.

## Supplementary information

Supplementary Information

Reporting Summary

## Data Availability

The microbial sequences and meta data generated in this study have been deposited in the MG-RAST database under accession code 17710. The monthly monitoring environmental data of Taihu Lake is available under restricted access according to data management policy and the access could be obtained from Taihu Laboratory for Lake Ecosystem Research (http://thl.cern.ac.cn/) or the corresponding author upon reasonable request. The other data are available under restricted access due to the authors’ continuing projects of field experiments on global mountainsides, and the access can be obtained from the corresponding author upon reasonable request.

## Code availability

The R codes for specialization metrics are available at 10.5281/zenodo.6592491, and at http://github.com/jianjunwang/iDOM.
