# Supplementary material for: Ecological networks of dissolved organic matter and microorganisms under global change
Source: Nat Commun. 2022 Jun 23;13:3600. doi: 10.1038/s41467-022-31251-1 (PMC9226077; doi:10.1038/s41467-022-31251-1)
Supplement: Supplementary file 1 — Supplementary Information [file 41467_2022_31251_MOESM1_ESM.pdf]

Supplementary Information for

Ecological networks of dissolved organic matter and microorganisms under global change

The file includes:

Supplementary Methods

Supplementary Tables (S1–S5)

Supplementary Figures (S1–S21)

Supplementary Methods

Considerations of the experimental design

In our study, we took advantage of elevational temperature gradients as a natural experiment. Natural climatic gradients along latitudinal and elevational gradients have played a pivotal role in testing the importance of climatic factors on biological community composition in the history of biogeography not only for macroorganisms, but also for microorganisms and biogeochemical cycling, and experimental manipulations along elevational or biogeographical gradients have been recognised as an important tool for disentangling different underlying drivers on community structure and ecosystem processes. For instance, De Sassi *et al.* [1] used a natural temperature gradient along elevations, combined with experimental nitrogen fertilization, to investigate the effects of elevated temperature and globally increasing anthropogenic nitrogen deposition on the structure and phenology of a grassland herbivore assemblage. Thus, using manipulative experiments along broad environmental gradients can help tease apart the relative importance of the interactive effects of local-scale factors and broad-scale climatic variation in shaping biological communities and ecosystem processes.

Temperature generally correlates strongly negatively with elevation, which was also true in our study showing significantly negatively correlation between measured water temperature and elevation in both China and Norway ($R^2 = 0.98$, $P < 0.05$, and $R^2 = 0.96$, $P < 0.05$, respectively). Specifically, we used the common sediment from Taihu Lake at each elevation with ten nutrient levels, and microbes from the local species pool of each elevation could be inoculated into the common sediment. As such, the covariance between climate and natural environments such as nutrients could be eliminated, which could disentangle the independent effects of temperature versus nutrient enrichment and also their interactive effects on the associations between DOM and microbes. Similar achievements are also reported in previous literature by experiments on mountainsides. For instance, in a translocation experiment, microbial decomposers were deliberately inoculated onto common leaf litter to disentangle the effects of microbial community versus temperature on litter decomposition rates and reevaluate the role of microbial community composition in its decomposition responses to climate [2].

However, for in-situ experiments, it is inevitable that there are some unmeasured environmental variables which might have some effects on biological communities and ecosystem processes. This will not be a major problem if our main focus was to investigate the major environmental variables which are playing pivotal roles in structuring biological and organic carbon compositions. Our data support that temperature and nutrients were key factors in explaining chemodiversity and bacterial community composition [3]. In our study, we considered as many important environmental variables as possible, such as chlorophyll *a*, bacterial abundance and nutrients. Due to practical and logistical issues, we were unable to measure all potential variables, such as UV radiation, across all elevations. This is largely because we have no clear evidence that these other variables have a dominant role in affecting the sediment bacterial and DOM compositions we studied here. More importantly, the bottom of our microcosm was buried into the local soils by 10% of the bottle height, and so sediments of each bottle were below the ground surface. It is thus unlikely that UV radiation and the related photodegradation would have stronger effects on bacteria and DOM in the sediments than temperature and nutrients.

In addition to relying on natural temperature gradients, controlled temperature experiments are another option to study climate effects on biological communities or organic matter decomposition. So far, there are two primary tools for simulating warming: passive greenhouses and active heating devices including soil, water and aerial arrays. Generally, controlling water temperatures in the field will encounter serious practical issues on mountainsides along large geographical regions. For instance, to prevent malfunction in a long-term run, heating devices are needed to be checked and replaced regularly, which is not possible in remote mountains and high elevations as in our current study. To obtain desired temperatures is also not easy in the field due to the variations such as in wind velocities, sun shine and forest shading along elevations. We do not see clearly how to practically and efficiently control for water temperatures in such a large-scale field study, and thus relied on natural temperature gradients.

It should be noted that our aims were considered to be as realistic as possible by combining field experiments and natural gradients, and by considering only the most

important and generally most interesting factors (that is, nutrients and temperature) for robust findings. Compared to field experiments, researchers can much more easily control virtually all aspects important for biological communities and organic matter in laboratory experiments. But laboratory experiments can be also easily criticized by some researchers to be far from realistic and results may not be applicable for real natural communities [4]. Different from laboratory manipulations, we think that these experiments are closer to nature environments, and expect such experiments to bridge between laboratory experiments and field observations. The communities in the microcosms should be dynamically driven by ecological processes, such as species dispersal, growth and extinction, and a time-series sampling would be ideally helpful to capture these processes. However, considering the harsh field situations in remote mountains or high elevations, the nature of destructive sampling, the large sample volumes required for repeat sampling, and the limited resources, it is unlikely we could have sampled biological communities and DOM at more than one time-point. As a tradeoff among logistics issue, heavy workload and robust results, we only sampled once after a one-month field incubation, and are confident that a final number of 300 DOM or bacterial samples across two mountains, 10 elevations and 10 nutrient levels was large enough for solid statistical analyses. We welcome future studies to undertake more intensive experiments if more resources could be allocated.

Supplementary Tables

Table S1. Variables used to explain the specialization of DOM-bacteria interactions. These variables were considered based on the hypothetical casual relationships in Fig. 6a: climate change, human impacts and contemporary nutrient variables as distal drivers, and energy supply, biodiversity, chemodiversity and DOM traits as proximal drivers. NMDS: non-metric multidimensional scaling.

Drivers	Group	Subgroup	Variable	Description
Distal drivers	Environment	Climate change	Temp	Water temperature[#1]
		Human impacts	ADD.NO_3	Nutrient enrichment[#2]
		Contemporary nutrient	TN.sedi	Sediment total nitrogen (TN)
		Contemporary nutrient	TP.sedi	Sediment total phosphorus (TP)
		Contemporary nutrient	NO_x.sedi	Sediment NO_x^-
		Contemporary nutrient	NO_2.sedi	Sediment NO_2^-
		Contemporary nutrient	NH_4.sedi	Sediment NH_4^+
		Contemporary nutrient	PO_4.sedi	Sediment PO_4^{3-}
		Contemporary nutrient	NO_3	Water NO_3^-
		Contemporary nutrient	NO_2	Water NO_2^-
		Contemporary nutrient	NH_4	Water NH_4^+
		Contemporary nutrient	PO_4	Water PO_4^{3-}
Proximal drivers	Energy supply	Energy supply	TC.sedi	Sediment total organic carbon (TOC)
		Energy supply	DOC.sedi	Sediment dissolved organic carbon (DOC)
		Energy supply	pH	Water pH
		Energy supply	Chla.sedi	Sediment Chlorophyll *a* (Chl *a*)
	Diversity	Biodiversity	bac.rich	Species richness of bacteria
		Biodiversity	bac.nmds1	NMDS axis 1 of bacterial composition
		Biodiversity	bac.nmds2	NMDS axis 2 of bacterial composition
		Chemodiversity	chemo.rich	Chemical richness of molecules
		Chemodiversity	mol.nmds1	NMDS axis 1 of molecular composition
		Chemodiversity	mol.nmds2	NMDS axis 2 of molecular composition
	Molecular traits	Molecular weight	Mass	The mass to charge ratio (m/z)
		Molecular weight	C	The number of carbon
		Molecular weight	$kdefect_{CH2}$	Kendrick Defect
		Stoichiometry	O/C	O/C ratio
		Stoichiometry	H/C	H/C ratio
		Stoichiometry	N/C	N/C ratio
		Stoichiometry	P/C	P/C ratio
		Stoichiometry	N/P	N/P ratio
		Stoichiometry	S/C	S/C ratio

	Chemical structure	AI_{Mod}	The modified aromaticity index
	Chemical structure	DBE	Double bond equivalence
	Chemical structure	DBE_{O}	Double bond equivalence minus oxygen
	Chemical structure	DBE_{AI}	Double bond equivalence minus aromaticity index
	Oxidation state	GFE	Gibbs free energy
	Oxidation state	NOSC	Nominal oxidation state of carbon
	Carbon use efficiency	Y_{met}	Carbon use efficiency

[#1] Water temperature was used to represent climatic variables due to its strong relations with elevation in both mountain ranges (Wang *et al*. 2016).

[#2] Nitrate addition ($ADD.NO_3$) was used to represent nutrient enrichment as the ratio between nitrate and phosphorus in the initial overlying water was constant.

Table S2. Variables used to explain DOM features. These variables were categorised into three groups: Environment (climate change, human impacts and contemporary nutrient), energy supply and biodiversity. NMDS: non-metric multidimensional scaling.

Group	Subgroup	Variable	Description
Environment	Climate change	Temp	Water temperature
	Human impacts	ADD.NO_3	Nutrient enrichment
	Contemporary nutrient	TN.sedi	Sediment total nitrogen (TN)
	Contemporary nutrient	TP.sedi	Sediment total phosphorus (TP)
	Contemporary nutrient	NO_x.sedi	Sediment NO_x^-
	Contemporary nutrient	NO_2.sedi	Sediment NO_2^-
	Contemporary nutrient	NH_4.sedi	Sediment NH_4^+
	Contemporary nutrient	PO_4.sedi	Sediment PO_4^{3-}
	Contemporary nutrient	NO_3	Water NO_3^-
	Contemporary nutrient	NO_2	Water NO_2^-
	Contemporary nutrient	NH_4	Water NH_4^+
	Contemporary nutrient	PO_4	Water PO_4^{3-}
Energy supply	Energy supply	TC.sedi	Sediment total organic carbon (TOC)
	Energy supply	DOC.sedi	Sediment dissolved organic carbon (DOC)
	Energy supply	pH	Water pH
	Energy supply	Chla.sedi	Sediment Chlorophyll *a* (Chl *a*)
Biodiversity	Biodiversity	bac.rich	Species richness of bacteria
	Biodiversity	bac.nmds1	NMDS axis 1 of bacterial composition
	Biodiversity	bac.nmds2	NMDS axis 2 of bacterial composition

Table S3. Formulae to calculate composite variables for structure equation models of the specialization H_2’ of DOM-bacteria interactions. We constructed four bipartite networks, that is, the negative and positive interaction networks in China or Norway. The obtained composite variables were used in Fig. 6. The abbreviations of included variables are listed in Table S1.

Response	Region	Network type	Composite	Formula
H_2'	China	Negative	Contemporary nutrient	-0.606 × NO_3 + -0.222 × NO_2 + 0.221 × PO_4 + -0.294 × TN.sedi + 0.184 × PO_4.sedi + 0.296 × NH_4.sedi + 0.219 × NO_2.sedi
			Energy supply	-0.589 × pH + 0.493× DOC.sedi
			Biodiversity	-0.278 × bac.nmds1 + 0.160 × bac.nmds2
			Chemodiversity	-0.451× chemo.rich + 0.481 × mol.nmds1
			Molecular traits	66.076 × Mass + -64.710 × C + -1.402 × AI_{Mod} + -15.166 × DBE + -20.154 × DBE_O + 61.402 × DBE_{AI} + -3.279 × GFE + -23.908 × $kdefect_{CH2}$ + -1.378 × O/C + 2.627 × H/C + 0.652 × N/P
H_2'	China	Positive	Contemporary nutrient	0.339 × NO_2 + -0.442 × PO_4 + 0.443 × PO_4.sedi + -0.173 × NO2.sedi
			Energy supply	0.525 × pH + 0.253 × Chla.sedi + -0.102 × TC.sedi + -0.262 × DOC.sedi
			Biodiversity	-0.293 × bac.rich + 0.718 × bac.nmds1
			Chemodiversity	-0.110 × chemo.rich + -0.591 × mol.nmds1 + 0.416 × mol.nmds2
			Molecular traits	-50.009 × Mass + 48.984 × C + 1.728 × AI_{Mod} + 3.075 × DBE_O + -26.770 × DBE_{AI} + 3.904 × GFE + 24.822 × $kdefect_{CH2}$ + 1.204 × O/C + -5.311 × H/C
H_2'	Norway	Negative	Contemporary nutrient	-0.381 × NO_3 + 0.365 × PO_4.sedi
			Energy supply	0.210 × DOC.sedi
			Chemodiversity	-0.359 × chemo.rich + -0.186 × mol.nmds1 + -0.120 × mol.nmds2
			Molecular traits	-1.316 × Mass + 1.062 × AI_{Mod} + 3.571 × DBE + 2.524 × DBE_O + -4.638 × DBE_{AI} + 2.193 × H/C + -0.387 × N/C + -0.372 × N/P
H_2'	Norway	Positive	Contemporary nutrient	-0.338 × NO_3 + 0.280 × NO_2 + 0.338 × PO_4 + 0.350 × PO_4.sedi + -0.105 × NO_2.sedi
			Energy supply	-0.327 × pH + 0.231 × TC.sedi + -0.350 × DOC.sedi
			Biodiversity	-0.510 × bac.nmds1
			Chemodiversity	-0.169 × mol.nmds1 + -0.480 × mol.nmds2
			Molecular traits	1.872 × Mass + -1.825 × DBE + 2.282 × DBE_{AI} + -2.674 × GFE + -1.431 × $kdefect_{CH2}$ + 1.288 × H/C + -0.877 × N/C + 1.653 × P/C

Table S4. Summary of the model fit statistics evaluated for standardized structural equation model (SEM). We explored the potential links between predictor variables and the specialization H_2’ of the negative and positive bipartite networks in China or Norway, and the best-fitting models are shown in Fig. 6. We constructed the full SEM models based on the hypothetical casual relationships (Fig. 6a), and further performed sequential models by dropping non-significant paths from the full models. χ^2: Chi-square. *P*: p-value of chi-square test. df: Degrees of freedom. CFI: Comparative fit index. SRMR: Standardized root mean squared residual. AICc: Second-order Akaike information criterion. ΔAICc: Delta AICc.

SEM models	Omitted paths	df	χ^2	*P*	CFI	SRMR	AICc	ΔAICc
**China; Negative**								
1[a]		1	0.563	0.453	1	0.004	1818.8	10.75
2	Human impacts -> H_2'	2	1.267	0.531	1	0.005	1816.6	8.53
3	Human impacts -> H_2'<br>Human impacts -> Chemodiversity	4	2.924	0.571	1	0.017	1812.5	4.46
4	Human impacts -> H_2'<br>Human impacts -> Chemodiversity<br>Human impacts -> Biodiversity	5	4.335	0.502	1	0.017	1811.2	3.08
5	Human impacts -> H_2'<br>Human impacts -> Chemodiversity<br>Human impacts -> Biodiversity<br>Human impacts -> Energy	6	6.201	0.401	1	0.017	1810.3	2.19
6	Human impacts -> H_2'<br>Human impacts -> Chemodiversity<br>Human impacts -> Biodiversity<br>Human impacts -> Energy<br>Nutrient -> DOM traits	7	8.631	0.280	1	0.018	1810.0	1.92
7	Human impacts -> H_2'<br>Human impacts -> Chemodiversity<br>Human impacts -> Biodiversity<br>Human impacts -> Energy<br>Nutrient -> DOM traits<br>Biodiversity -> DOM traits	8	10.579	0.227	1	0.020	1809.3	1.20
8[b]	Human impacts -> H_2'<br>Human impacts -> Chemodiversity<br>Human impacts -> Biodiversity<br>Human impacts -> Energy<br>Nutrient -> DOM traits<br>Biodiversity -> DOM traits<br>Climate change -> DOM traits	7	6.714	0.459	1	0.018	1808.1	0
**China; Positive**								
1[a]		1	0.760	0.383	1	0.004	1966.3	15.04

2	Nutrient -> Biodiversity	2	0.768	0.681	1	0.004	1963.4	12.11
3	Nutrient -> Biodiversity<br>Energy -> H_2'	3	0.869	0.833	1	0.004	1960.6	9.33
4	Nutrient -> Biodiversity<br>Energy -> H_2'<br>Human impacts -> H_2'	4	1.086	0.896	1	0.005	1958.0	6.71
5	Nutrient -> Biodiversity<br>Energy -> H_2'<br>Human impacts -> H_2'<br>Nutrient -> DOM traits	5	1.477	0.916	1	0.006	1955.6	4.31
6	Nutrient -> Biodiversity<br>Energy -> H_2'<br>Human impacts -> H_2'<br>Nutrient -> DOM traits<br>Human impacts -> Biodiversity	6	2.526	0.866	1	0.009	1953.9	2.61
7	Nutrient -> Biodiversity<br>Energy -> H_2'<br>Human impacts -> H_2'<br>Nutrient -> DOM traits<br>Human impacts -> Biodiversity<br>Climate change -> H_2'	7	3.888	0.793	1	0.010	1952.6	1.26
8[b]	Nutrient -> Biodiversity<br>Energy -> H_2'<br>Human impacts -> H_2'<br>Nutrient -> DOM traits<br>Human impacts -> Biodiversity<br>Climate change -> H_2'<br>Climate change -> Chemodiversity	8	5.291	0.726	1	0.013	1951.3	0
9	Nutrient -> Biodiversity<br>Energy -> H_2'<br>Human impacts -> H_2'<br>Nutrient -> DOM traits<br>Human impacts -> Biodiversity<br>Climate change -> H_2'<br>Climate change -> Chemodiversity<br>Human impacts -> DOM traits	9	8.220	0.512	1	0.019	1951.6	0.30
10	Nutrient -> Biodiversity<br>Energy -> H_2'<br>Human impacts -> H_2'<br>Nutrient -> DOM traits<br>Human impacts -> Biodiversity<br>Climate change -> H_2'<br>Climate change -> Chemodiversity<br>Human impacts -> DOM traits<br>Nutrient -> H_2'	10	11.134	0.347	1	0.023	1951.9	0.63
**Norway; Negative**								
1[a]		0	0	0	1	0	1225.2	11.51
2	Energy -> Chemodiversity	1	0.120	0.729	1	0.004	1222.6	8.96

3	Energy -> Chemodiversity<br>Climate change -> Energy	2	0.263	0.877	1	0.006	1220.1	6.48
4	Energy -> Chemodiversity<br>Climate change -> Energy<br>Energy -> H_2'	3	0.656	0.884	1	0.007	1218.0	4.29
5	Energy -> Chemodiversity<br>Climate change -> Energy<br>Energy -> H_2'<br>Nutrient -> DOM traits	4	1.097	0.895	1	0.011	1215.8	2.18
6	Energy -> Chemodiversity<br>Climate change -> Energy<br>Energy -> H_2'<br>Nutrient -> DOM traits<br>Climate change -> Nutrient	5	2.097	0.836	1	0.022	1214.3	0.67
7[b]	Energy -> Chemodiversity<br>Climate change -> Energy<br>Energy -> H_2'<br>Nutrient -> DOM traits<br>Climate change -> Nutrient<br>Climate change -> H_2'	6	3.893	0.691	1	0.024	1213.7	0
8	Energy -> Chemodiversity<br>Climate change -> Energy<br>Energy -> H_2'<br>Nutrient -> DOM traits<br>Climate change -> Nutrient<br>Climate change -> H_2'<br>Chemodiversity -> DOM traits	7	6.735	0.457	1	0.038	1214.1	0.41
**Norway; Positive**								
1[a]		0	0	0	1	0	1751.2	19.79
2	Human impacts -> Chemodiversity	1	0.097	0.756	1	0.002	1748.3	16.91
3	Human impacts -> Chemodiversity<br>Biodiversity -> DOM traits	2	0.225	0.894	1	0.002	1745.5	14.11
4	Human impacts -> Chemodiversity<br>Biodiversity -> DOM traits<br>Energy -> DOM traits	3	0.391	0.942	1	0.003	1742.8	11.39
5	Human impacts -> Chemodiversity<br>Biodiversity -> DOM traits<br>Energy -> DOM traits<br>Energy -> H_2'	4	0.802	0.938	1	0.006	1740.4	8.97
6	Human impacts -> Chemodiversity<br>Biodiversity -> DOM traits<br>Energy -> DOM traits<br>Energy -> H_2'<br>Climate change -> H_2'	5	1.286	0.936	1	0.008	1738.0	6.66

7	Human impacts -> Chemodiversity<br>Biodiversity -> DOM traits<br>Energy -> DOM traits<br>Energy -> H_2'<br>Climate change -> H_2'<br>Human impacts -> Biodiversity	6	1.892	0.929	1	0.009	1735.9	4.51
8	Human impacts -> Chemodiversity<br>Biodiversity -> DOM traits<br>Energy -> DOM traits<br>Energy -> H_2'<br>Climate change -> H_2'<br>Human impacts -> Biodiversity<br>Nutrient -> Energy	7	2.513	0.926	1	0.011	1733.8	2.43
9	Human impacts -> Chemodiversity<br>Biodiversity -> DOM traits<br>Energy -> DOM traits<br>Energy -> H_2'<br>Climate change -> H_2'<br>Human impacts -> Biodiversity<br>Nutrient -> Energy<br>Chemodiversity -> H_2'	8	3.949	0.862	1	0.012	1732.6	1.2
10[b]	Human impacts -> Chemodiversity<br>Biodiversity -> DOM traits<br>Energy -> DOM traits<br>Energy -> H_2'<br>Climate change -> H_2'<br>Human impacts -> Biodiversity<br>Nutrient -> Energy<br>Chemodiversity -> H_2'<br>Nutrient -> H_2'	9	5.377	0.800	1	0.013	1731.4	0
11	Human impacts -> Chemodiversity<br>Biodiversity -> DOM traits<br>Energy -> DOM traits<br>Energy -> H_2'<br>Climate change -> H_2'<br>Human impacts -> Biodiversity<br>Nutrient -> Energy<br>Chemodiversity -> H_2'<br>Nutrient -> H_2'<br>Human impacts -> DOM traits	10	8.458	0.584	1	0.017	1731.9	0.5

[a] Full SEM models; [b] Best-fitting models shown in red.

Table S5. The hypothesized causal relationships and path coefficients in the structural equation model (Fig. 6a).

Relationship	Path coefficients
Climate change -> Nutrient	$\lambda_{nut,temp}$
Human impacts -> Nutrient	$\lambda_{nut,N}$
Climate change -> Energy	$\lambda_{energy,temp}$
Human impacts -> Energy	$\lambda_{energy,N}$
Nutrient -> Energy	$\lambda_{energy,nut}$
Climate change -> Biodiversity	$\lambda_{biodiv,temp}$
Human impacts -> Biodiversity	$\lambda_{biodiv,N}$
Nutrient -> Biodiversity	$\lambda_{biodiv,nut}$
Energy -> Biodiversity	$\lambda_{biodiv,energy}$
Climate change -> Chemodiversity	$\lambda_{chemodiv,temp}$
Human impacts -> Chemodiversity	$\lambda_{chemodiv,N}$
Nutrient -> Chemodiversity	$\lambda_{chemodiv,nut}$
Energy -> Chemodiversity	$\lambda_{chemodiv,energy}$
Climate change -> DOM traits	$\lambda_{trait,temp}$
Human impacts -> DOM traits	$\lambda_{trait,N}$
Nutrient -> DOM traits	$\lambda_{trait,nut}$
Energy -> DOM traits	$\lambda_{trait,energy}$
Biodiversity -> DOM traits	$\lambda_{trait,biodiv}$
Chemodiversity -> DOM traits	$\lambda_{trait,chemodiv}$
Climate change -> H_2'	$\lambda_{H2,temp}$
Human impacts -> H_2'	$\lambda_{H2,N}$
Nutrient -> H_2'	$\lambda_{H2,nut}$
Energy -> H_2'	$\lambda_{H2,energy}$
Biodiversity -> H_2'	$\lambda_{H2,biodiv}$
Chemodiversity -> H_2'	$\lambda_{H2,chemodiv}$
DOM traits -> H_2'	$\lambda_{H2,trait}$

Supplementary Figures

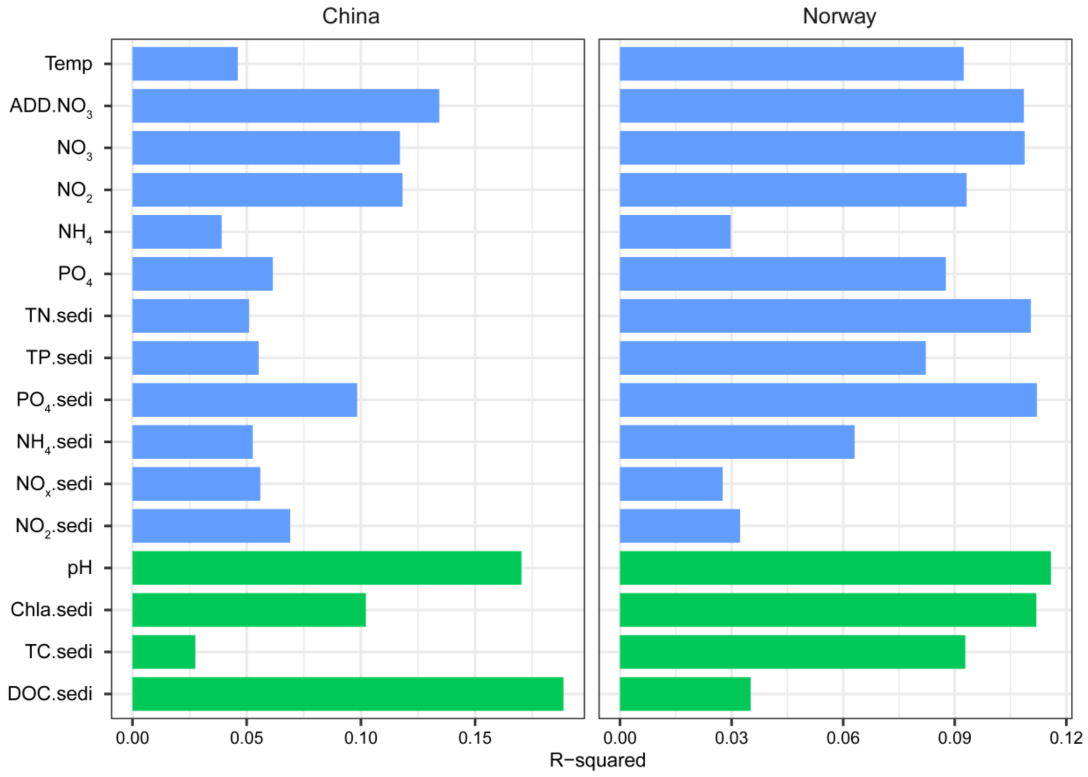

Figure S1. Bar plots showing R-squared to identify the effects of environmental (blue) and energy supply (green) variables on molecular composition of DOM in China and Norway. R-squared was determined by permutational multivariate analysis of variance (PERMANOVA) with 999 permutations and was statistically significant ($P \leq 0.001$) for explanatory variables. R-squared for each explanatory variable stands for the explained variations in the differences of DOM molecular composition among the samples. Nitrate addition (ADD.NO_3) was used to represent nutrient enrichment as the ratio between nitrate and phosphorus in the initial overlying water was constant. The abbreviations of explanatory variables are detailed in Table S2.

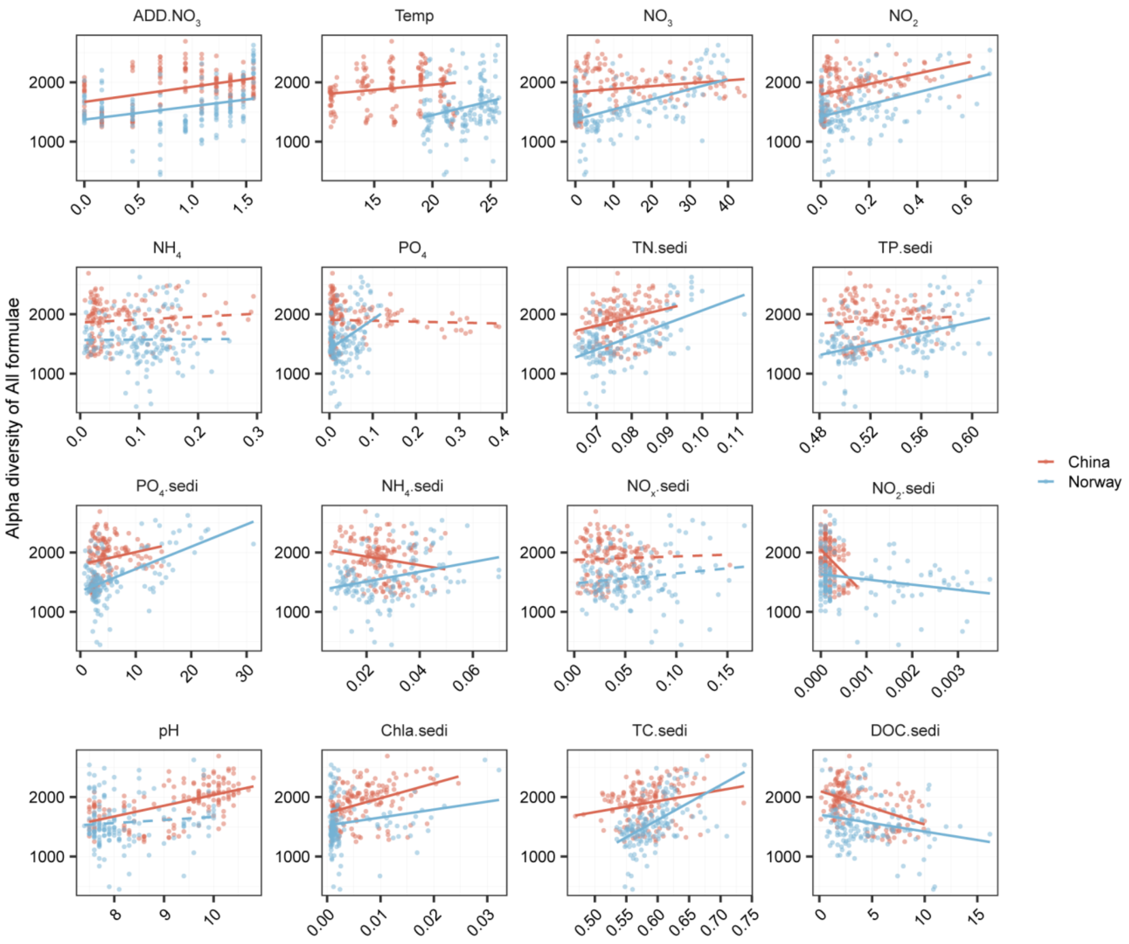

Figure S2. The relationships between DOM alpha diversity (i.e., molecular richness) and explanatory variables in China (red lines) and Norway (blue lines). We plotted alpha diversity against the variables relevant to environment and energy supply (Table S2). The relationships are indicated by solid ($P \leq 0.05$) and dotted ($P > 0.05$) lines estimated using linear models with one-sided F-statistics.

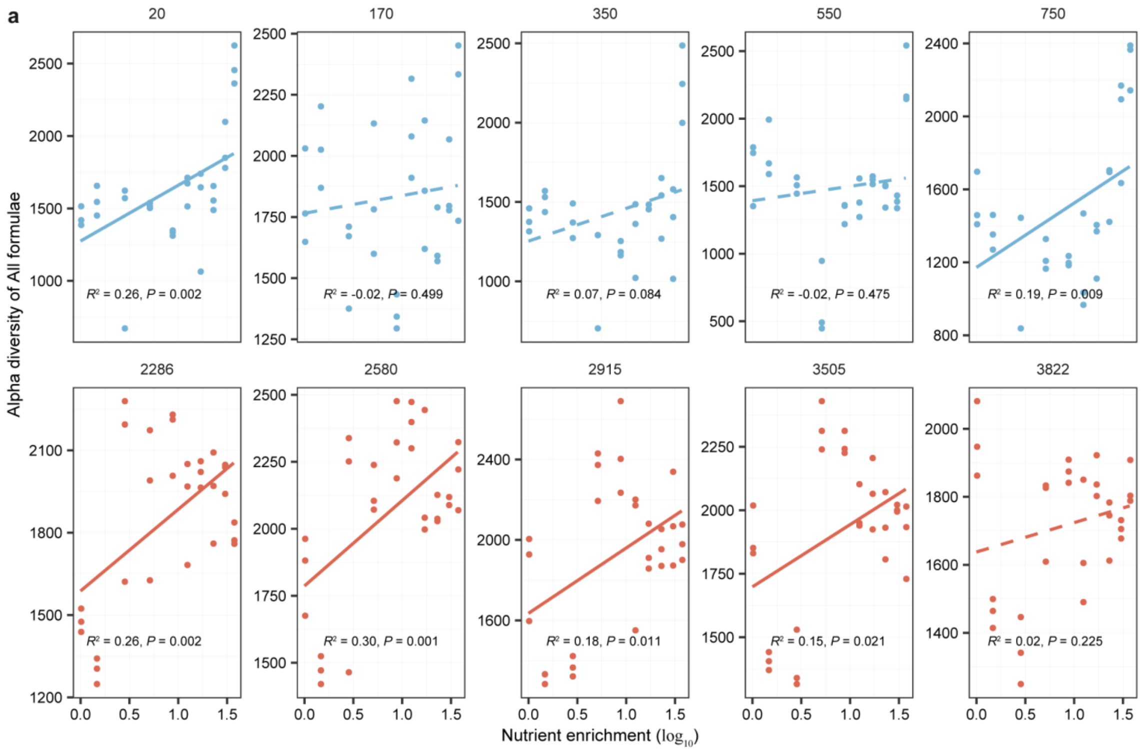

Figure S3. The relationships between nutrient enrichment and DOM alpha diversity or molecular traits in China (red lines) and Norway (blue lines) at different elevations (20 to 3,822 m a.s.l.). We considered richness for all formulae (a) and also molecular traits such as weighted means of mass (b), H/C ratio (c), O/C ratio (d) and AI_{Mod} (e) in China (red lines and dots) and Norway (blue lines and dots). We used nitrate addition to represent nutrient enrichment as the ratio between nitrate and phosphorus in the initial overlying water was constant. We plotted the richness or traits against the nutrient gradient of nitrate, and their relationships are indicated by solid ($P \leq 0.05$) and dotted ($P > 0.05$) lines using linear models with one-sided F-statistics.

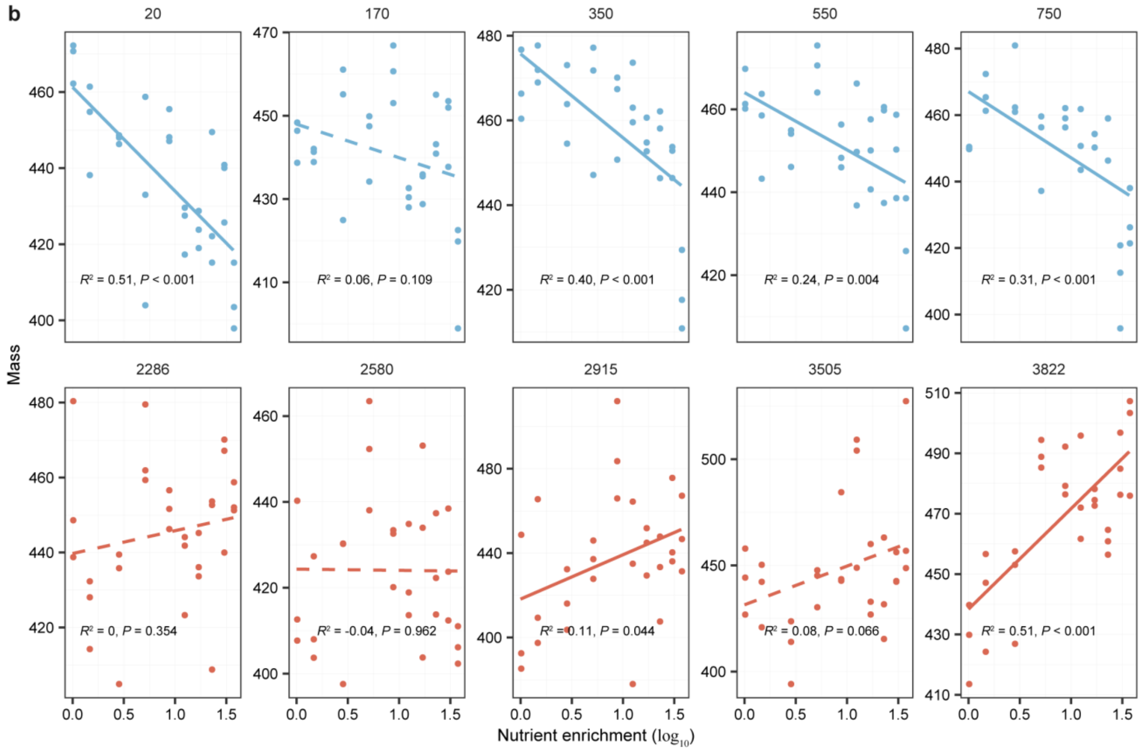

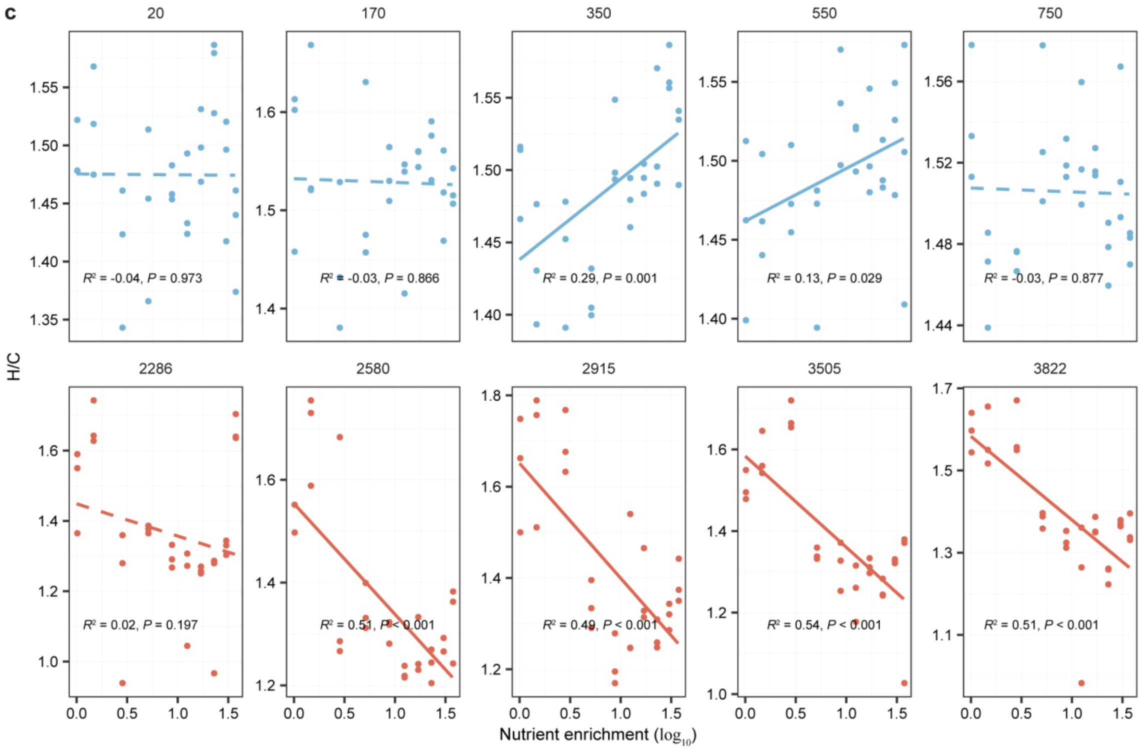

Figure S3. Continued. Weighted means of mass (b), and H/C ratio (c).

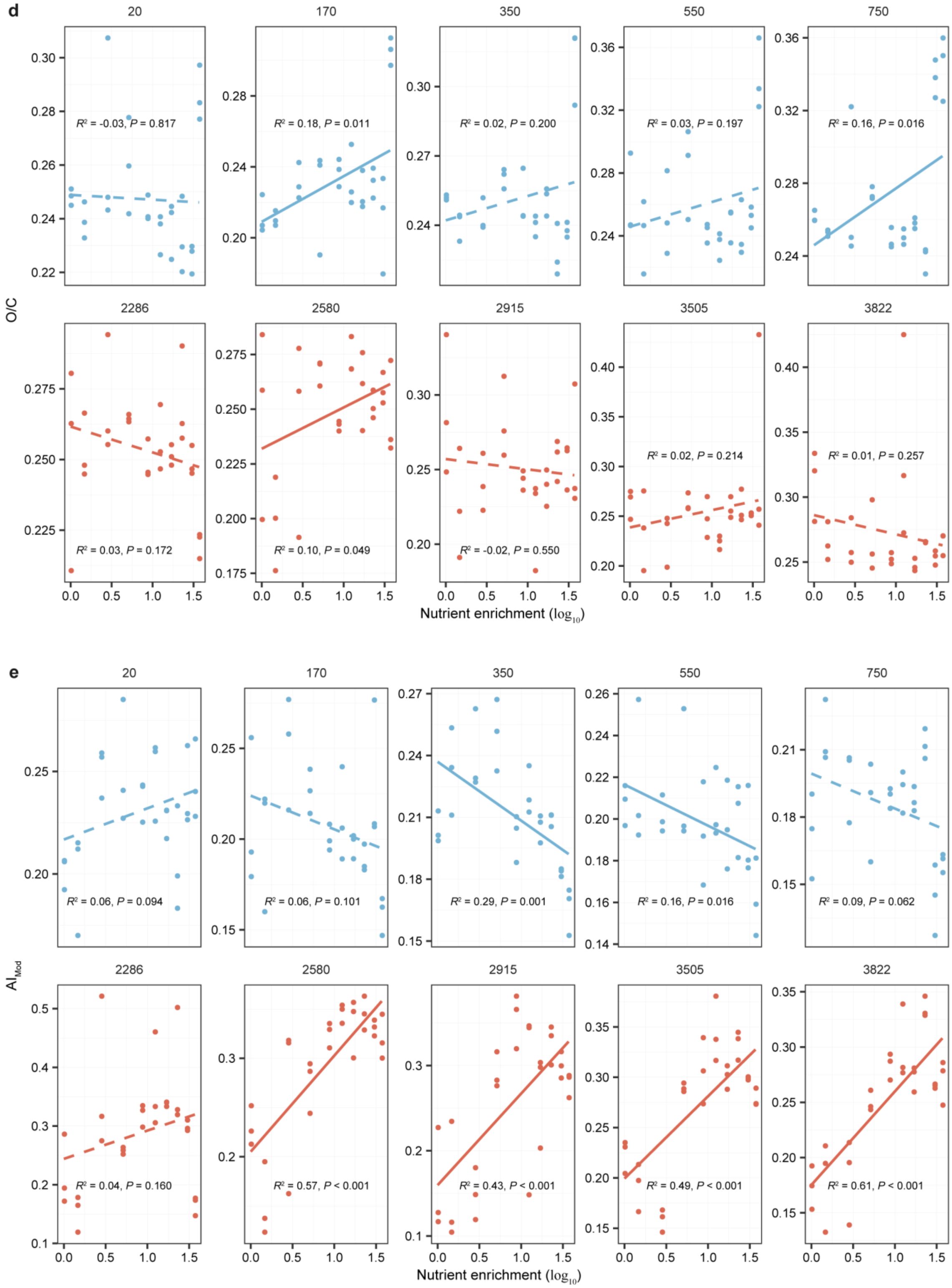

Figure S3. Continued. Weighted means of O/C ratio (d) and AI_{Mod} (e).

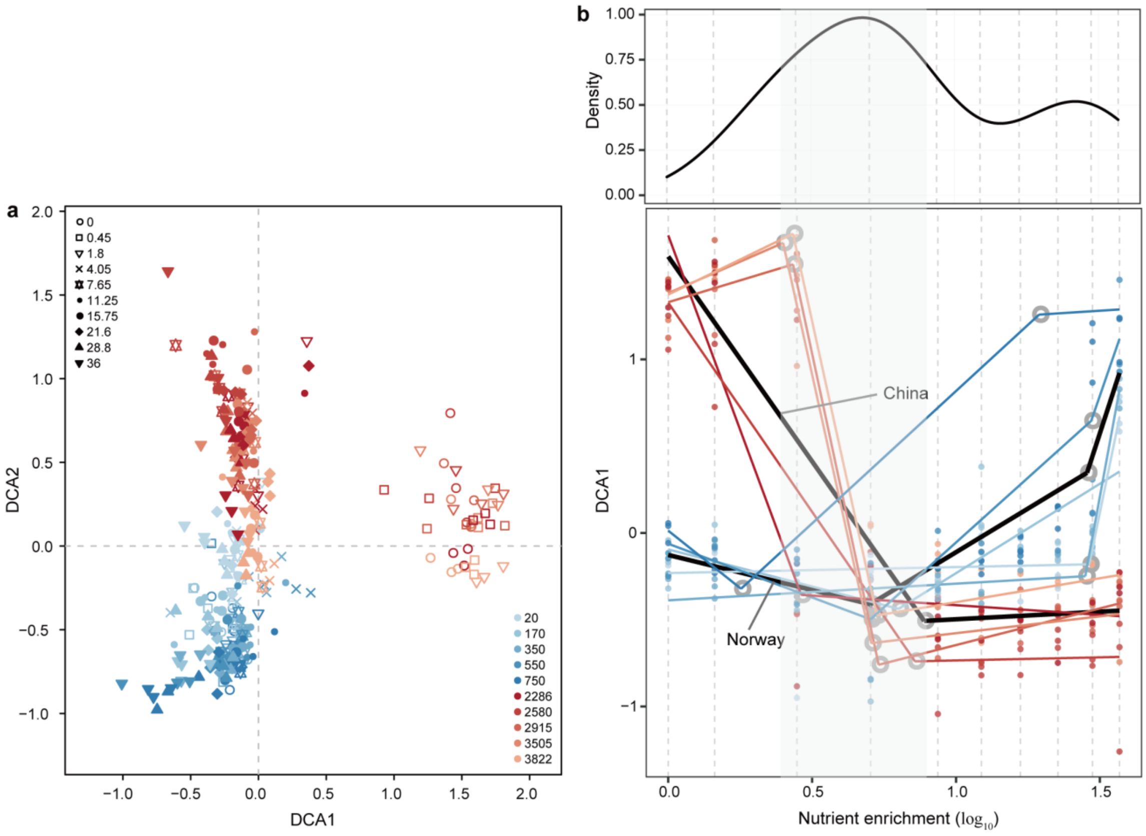

Figure S4. Variations in DOM compositions along the nutrient gradient of nitrate. (a) Detrended correspondence analyses (DCA) of DOM compositions. (b) Nutrient breakpoint estimation of the first axis of the DCA of DOM composition for each elevation using piecewise regression analysis with Bayesian Information Criteria statistics (Muggeo, 2008). The upper panel is a density plot of the distribution of breakpoints. The black thick lines in the lower panel indicate each region (i.e., China and Norway), and the colored dots or lines indicate the elevations of the two regions, which are consistent with the figure legend of Fig. S4a. The gray open circles indicate nutrient breakpoints. The vertical gray lines indicate the ten experimental nutrient levels. We found that, along the nutrient gradient, there were breakpoints of the first axis of the DCA mostly occurring between 1.80 and 4.05 mg N L^{-1} (indicated by gray shade) especially in China.

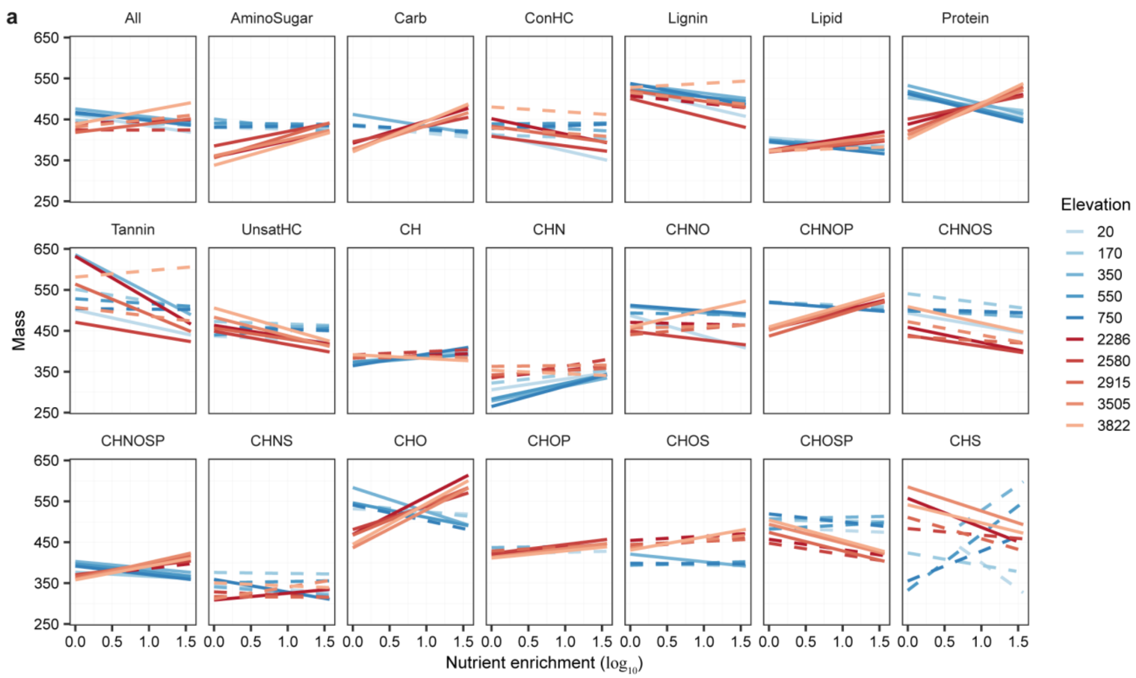

Figure S5. Effects of nutrient enrichment on DOM traits for all formulae and subsets of formulae within compound classes or elemental combinations across different elevations in China (red lines) and Norway (blue lines). We considered molecular traits such as weighted means of mass (a), H/C ratio (b) and AI_{Mod} (c), which were plotted against the nutrient gradient of nitrate, and their relationships are indicated by the solid ($P \leq 0.05$) or dotted ($P > 0.05$) lines using linear models with one-sided F-statistics. The details of abbreviations of DOM traits are available in Table S1. We found that nutrient enrichment increased the weighted means of molecular mass more strongly at higher elevations in China (with maximal 495 Da at 3,822 m a.s.l.), but decreased more strongly at lower elevations in Norway (with minimal 405 Da at 20 m a.s.l.). This finding implies that nutrient enrichment leads to an increase in the molecular mass especially at colder temperatures in subtropical regions, but a decline at the warmer temperatures in subarctic regions.

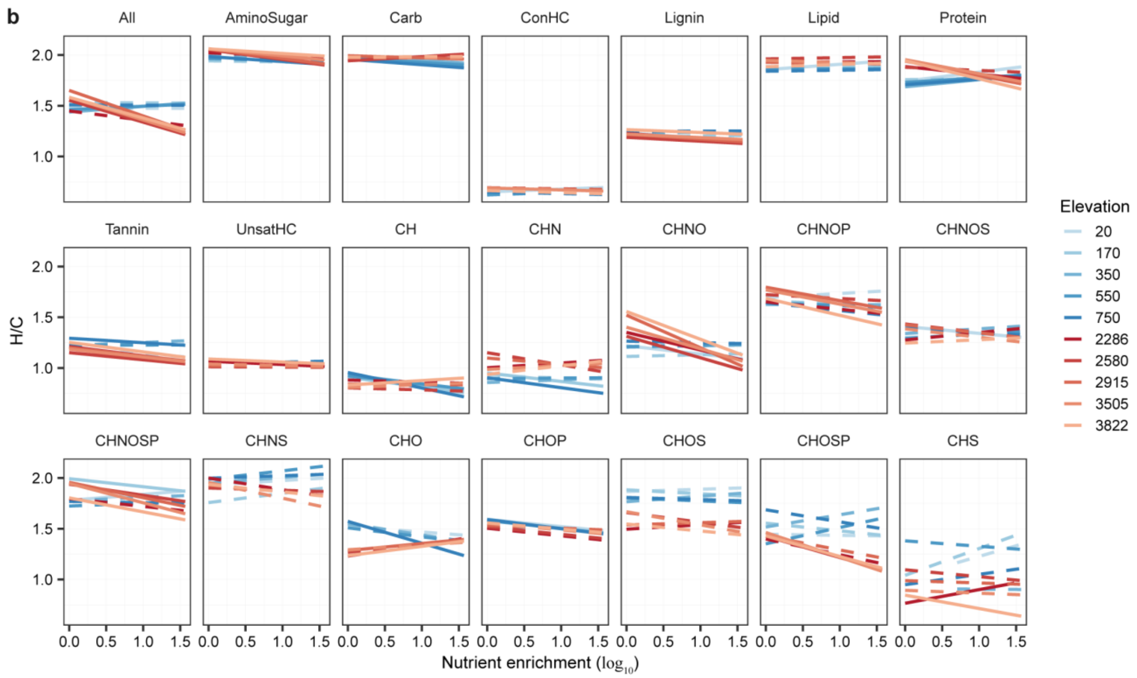

Figure S5. Continued. Weighted means of H/C ratio (b).

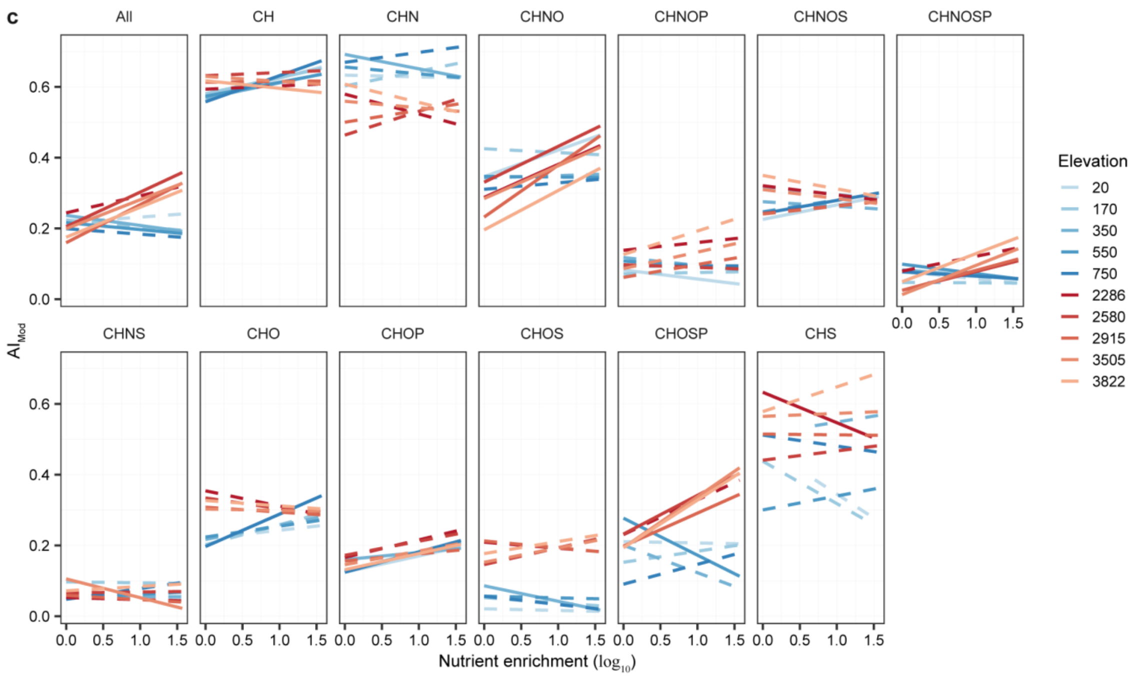

Figure S5. Continued. Weighted means of AI_{Mod} (c).

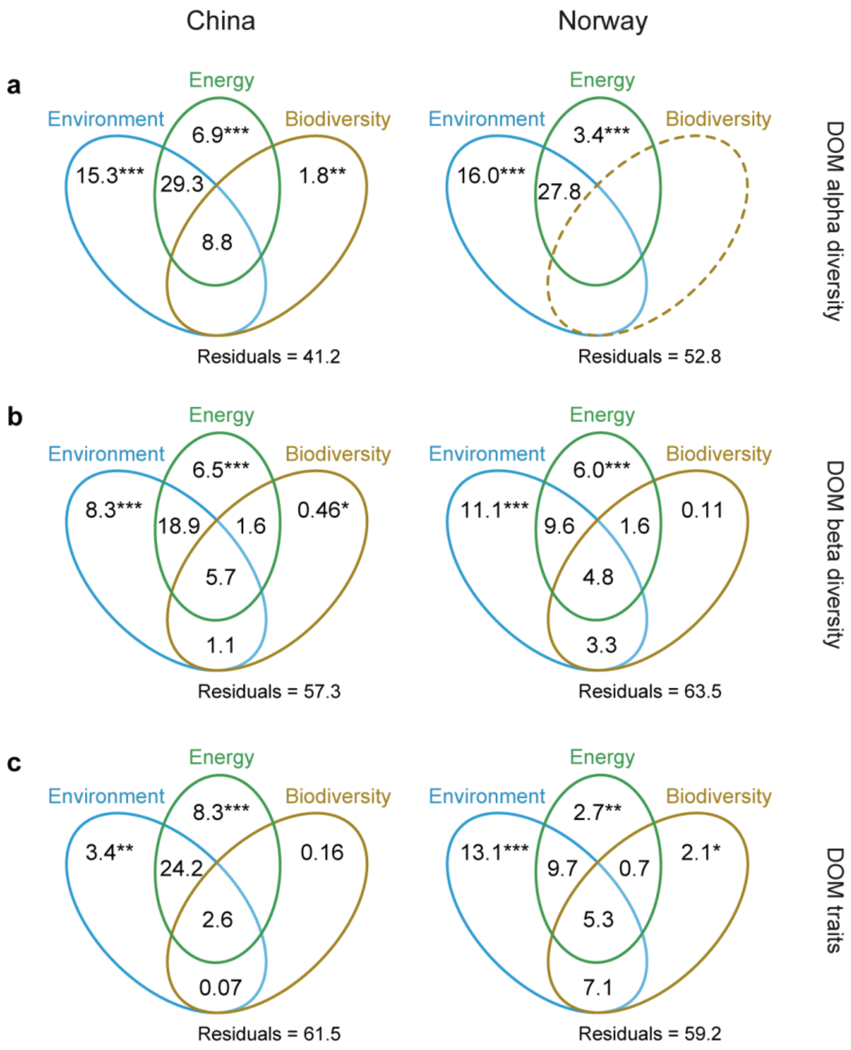

Figure S6. The roles of microbes in explaining the alpha diversity (upper panel), beta diversity (middle panel) and molecular traits (lower panel) of DOM using variation partitioning analysis. The numbers indicate the variance explained (%) by environments, energy supply and bacterial biodiversity which were described in detail in Table S2. The significance was examined using one-sided F-statistics and asterisks represent statistically significant effects at ***, $P \leq 0.001$; **, $P \leq 0.01$; *, $P \leq 0.05$.

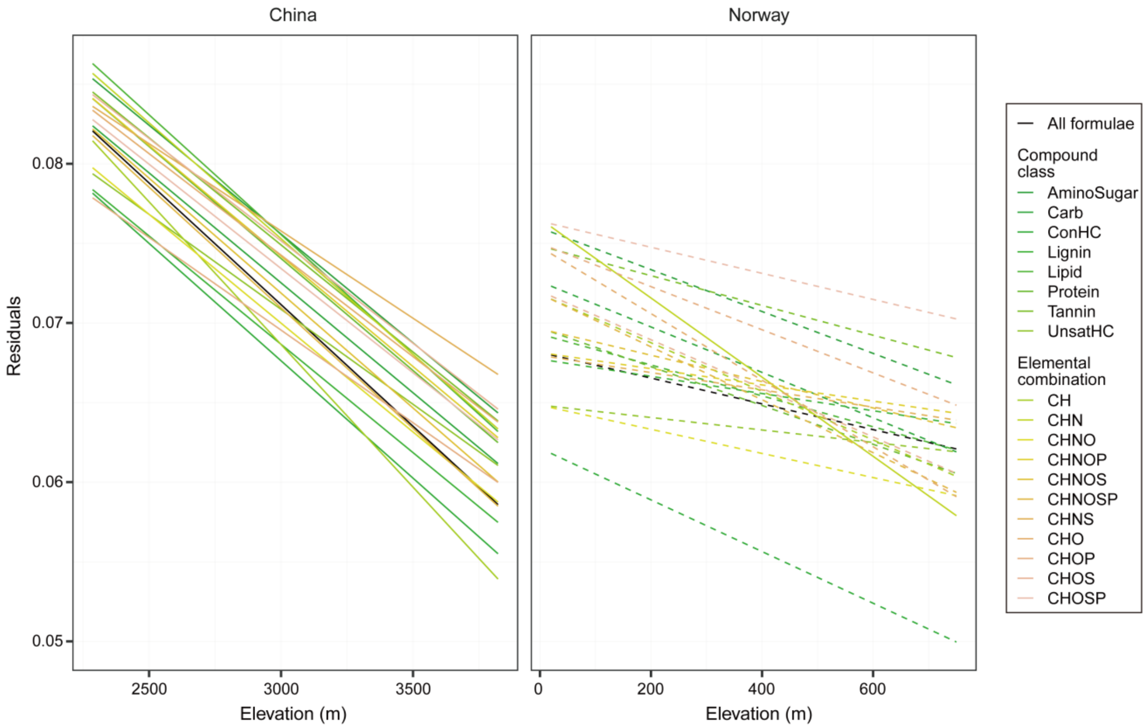

Figure S7. The effects of elevation on DOM-microbe associations indicated by Procrustes residuals, that is, the difference in composition between DOM and bacteria for each microcosm. These effects are indicated by solid ($P \leq 0.05$) or dotted ($P > 0.05$) lines estimated using linear models with one-sided F-statistics. The colours of the lines indicate the DOM composition for all formulae and categories of compound classes or elemental combinations.

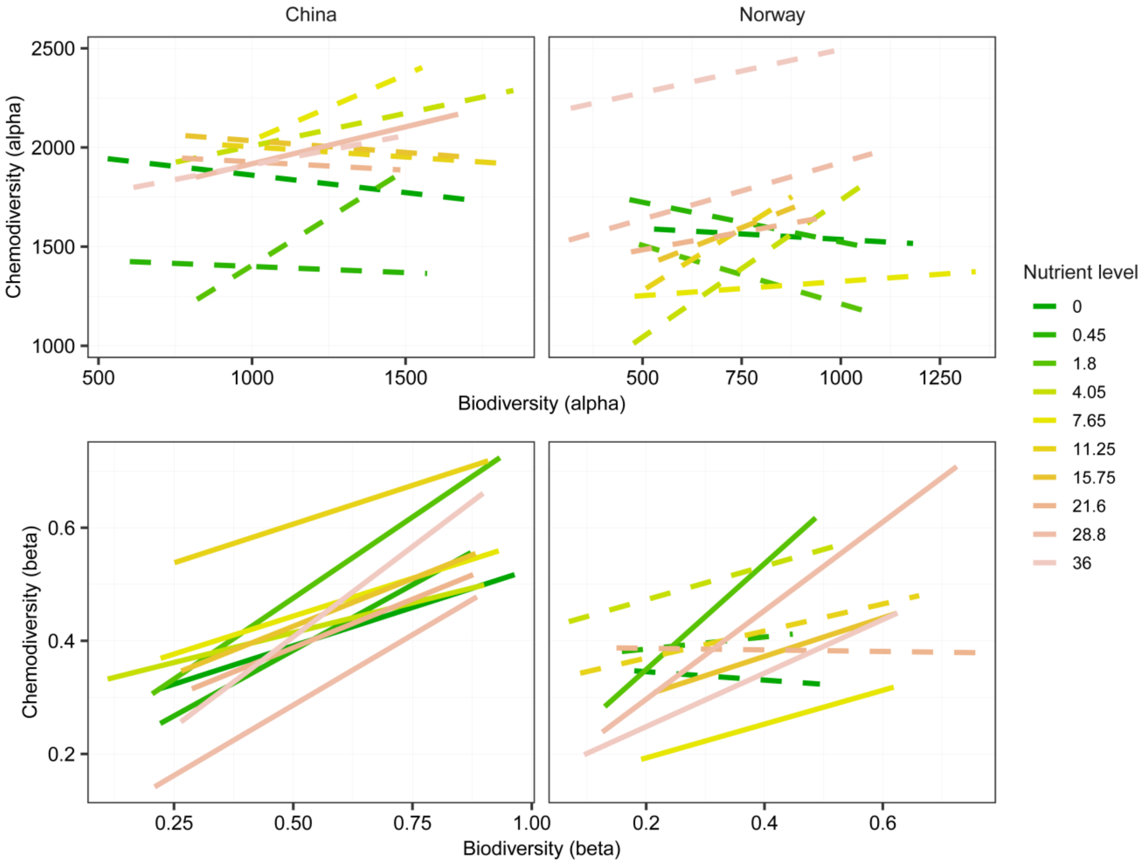

Figure S8. The relationships between chemodiversity and bacterial diversity in China and Norway along the nutrient gradient of nitrate. Upper panel: Alpha diversity (richness) of DOM molecular formulae and bacterial OTUs. Lower panel: Beta diversity determined by the Bray–Curtis dissimilarity index for the mixtures of molecular formulae and the communities of OTUs. Each line visualises the relationship of alpha or beta diversity between DOM and bacteria across 15 samples at each nutrient level in China or Norway. The relationships are indicated by solid ($P \leq 0.05$) and dotted ($P > 0.05$) lines using linear models, and the significance was determined by ANOVA with one-sided F-statistics (upper panels) or two-sided Mantel test with 999 permutations (lower panels).

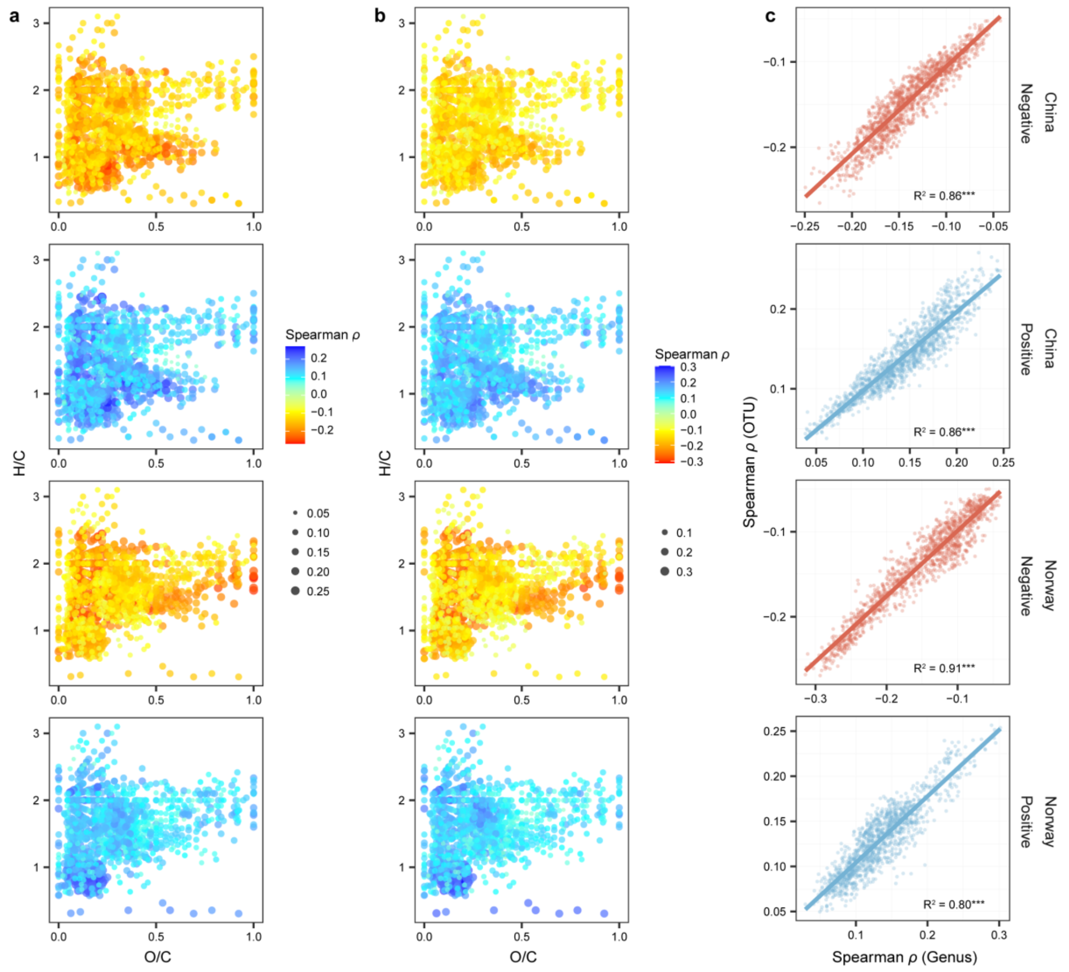

Figure S9. Molecular formulae correlating with bacterial OTUs (a) or genera (b) in China and Norway using Spearman's rank correlation (ρ). Each molecule is colored by the mean ρ value of negative or positive correlations across all bacterial OTUs, and the absolute value of mean ρ is indicated by the dot size. (c) The relationships in Spearman ρ between bacterial OTU and genus levels for both negative and positive correlations in China and Norway. Solid lines indicate significant linear fits with one-sided F-statistics ($P \leq 0.05$).

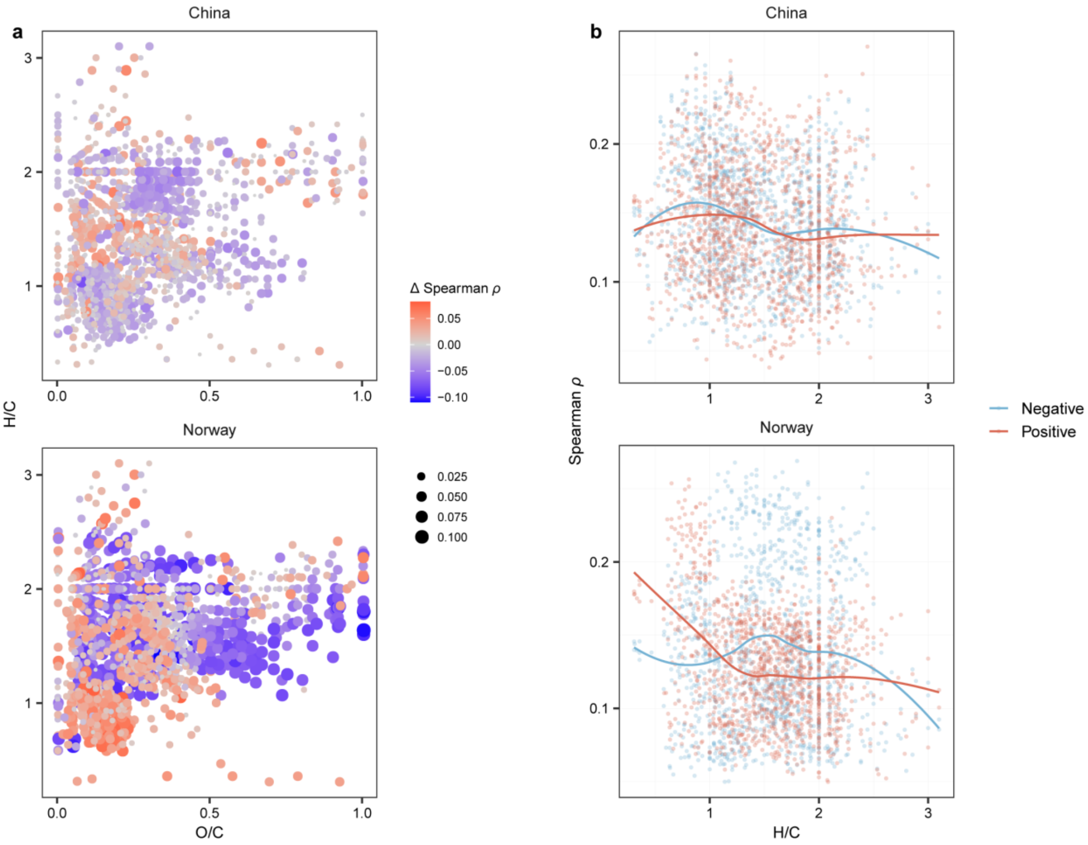

Figure S10. Correlations between DOM and bacteria regarding molecular traits. (a) Molecular formulae correlating with bacterial OTUs in China and Norway using Spearman's rank correlation (ρ). Each molecule is colored by difference in the absolute Spearman ρ ($\Delta\rho$). The difference for each molecule was calculated by subtracting the mean absolute ρ value of the negative correlations across all bacterial OTUs from that of the negative correlations, and the absolute value of ρ difference is indicated by the dot size. (b) The patterns of absolute ρ values of positive and negative correlations along the gradient of H/C ratio visualized with loess regression models.

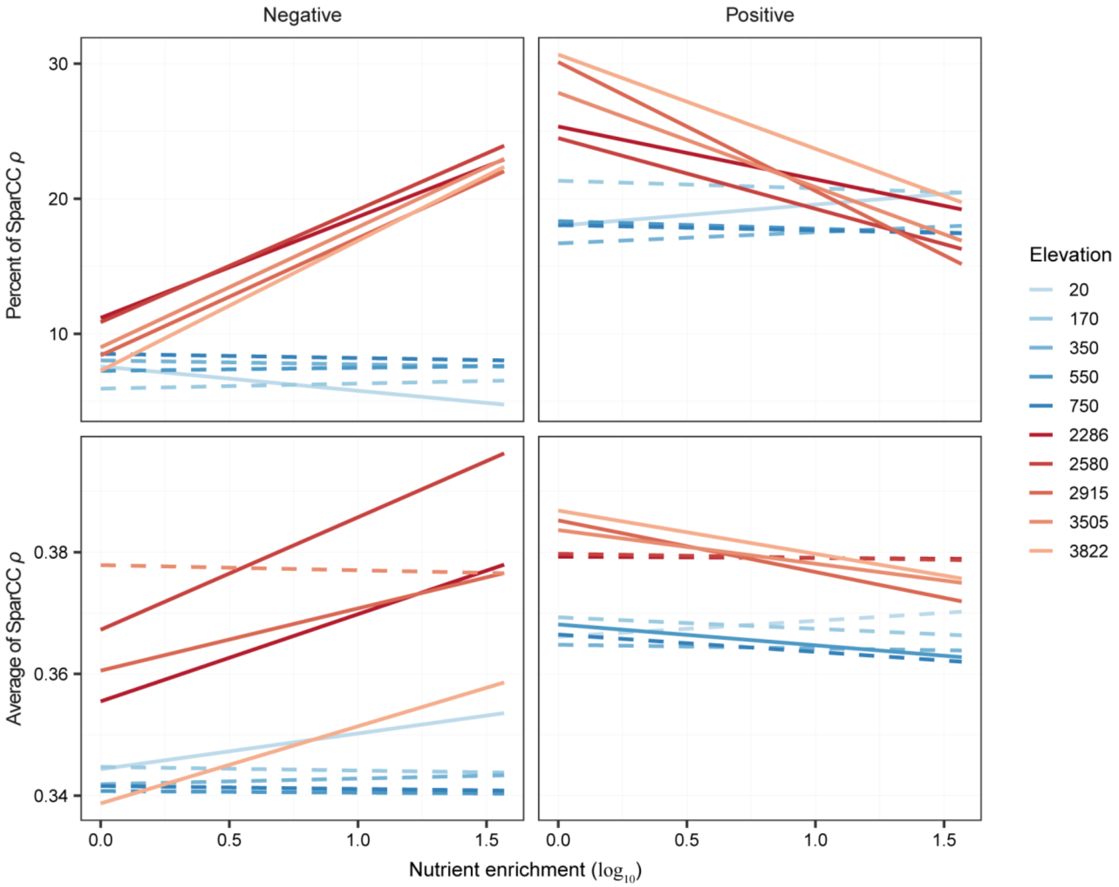

Figure S11. The effects of nutrient enrichment on weighted means of indices of DOM-bacteria bipartite networks. The network indices include the percent and average of strong correlations (|SparCC ρ| ≥ 0.3) of negative or positive networks. We plotted these indices against the nutrient gradient of nitrate for both negative (left panel) and positive (right panel) networks for each elevation in China (red lines) or Norway (blue lines), and their relationships are indicated by solid ($P \leq 0.05$) or dotted ($P > 0.05$) lines using linear models with one-sided F-statistics.

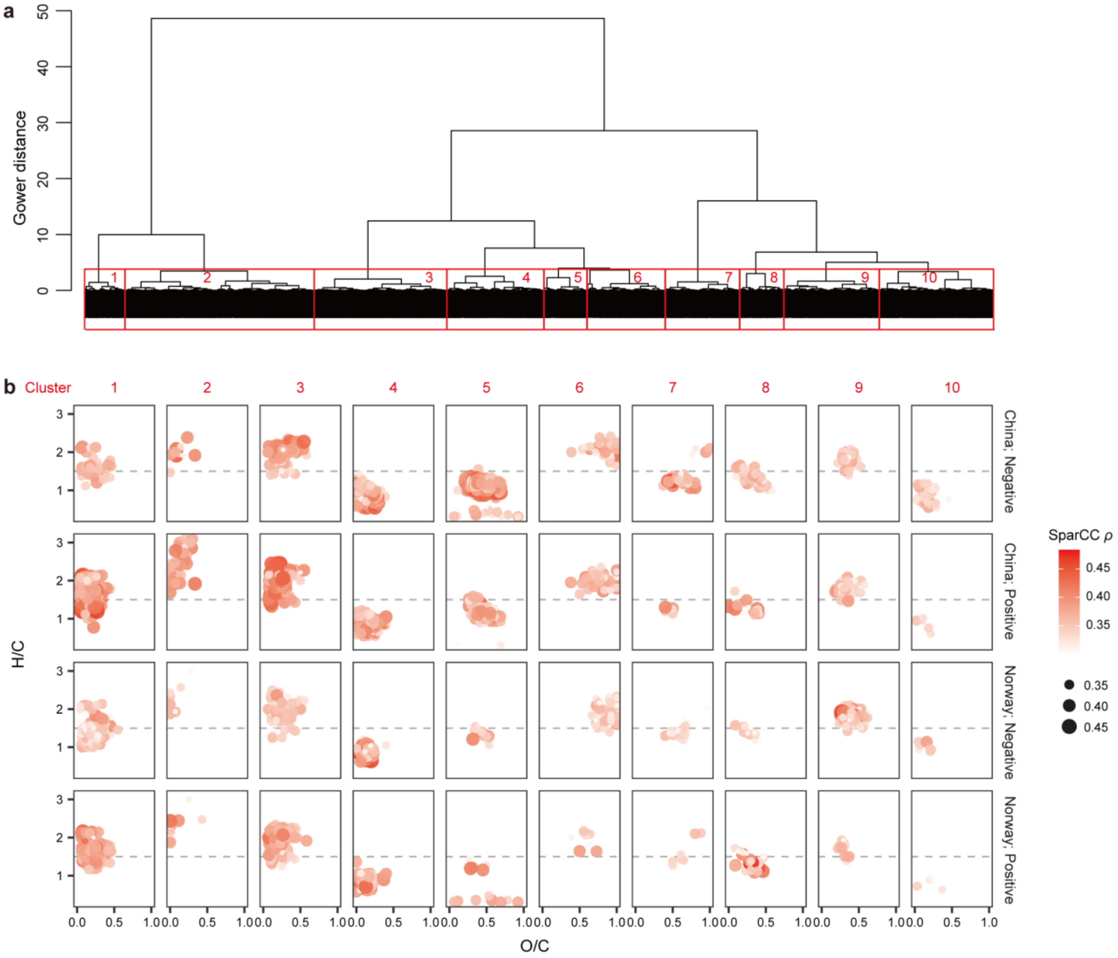

Figure S12. Correlations between DOM and bacteria regarding molecular traits. (a) Cluster analysis identified ten molecular sub-mixtures based on 16 molecular traits (Table S1). (b) Location of the ten clusters in Van Krevelen space with colour- and size-coded correlations between molecule-specific intensities and the relative abundance of bacterial genera using SparCC (Sparse Correlations for Compositional data). For each molecule, we showed the mean absolute SparCC ρ values of negative or positive correlations across all bacterial OTUs. We considered only strong correlations (|SparCC ρ| $\geq$ 0.3).

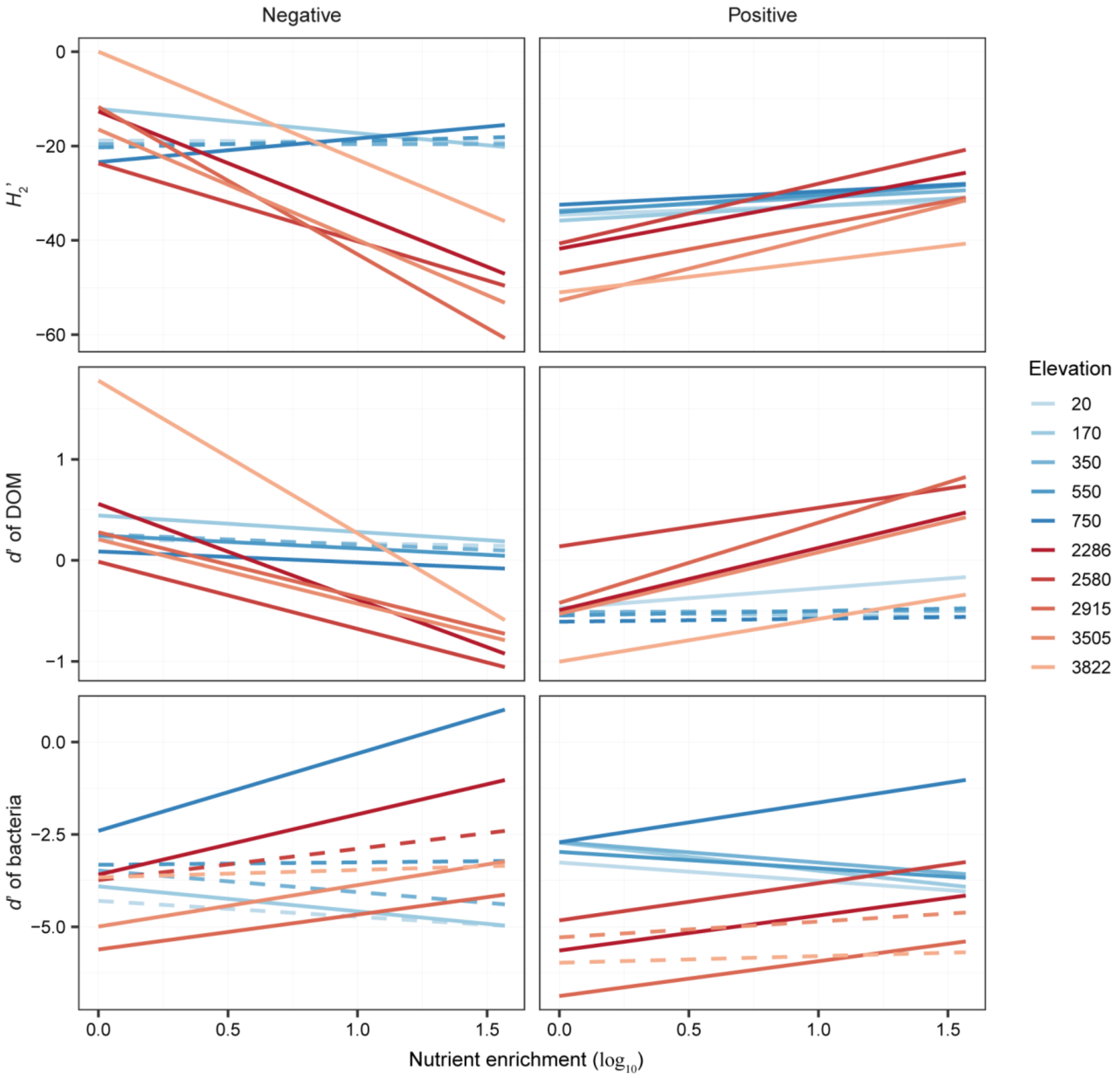

Figure S13. The effects of nutrient enrichment on specialization indices of DOM-bacteria bipartite networks. The specialization indices include network-level specialization H_2', and the weighted means of specialization *d*' for DOM molecules and bacterial genera. We plotted these indices against the nutrient gradient of nitrate for both negative (left panel) and positive (right panel) networks for each elevation in China (red lines) or Norway (blue lines), and their relationships are indicated by solid ($P \leq 0.05$) or dotted ($P > 0.05$) lines using linear models with one-sided F-statistics.

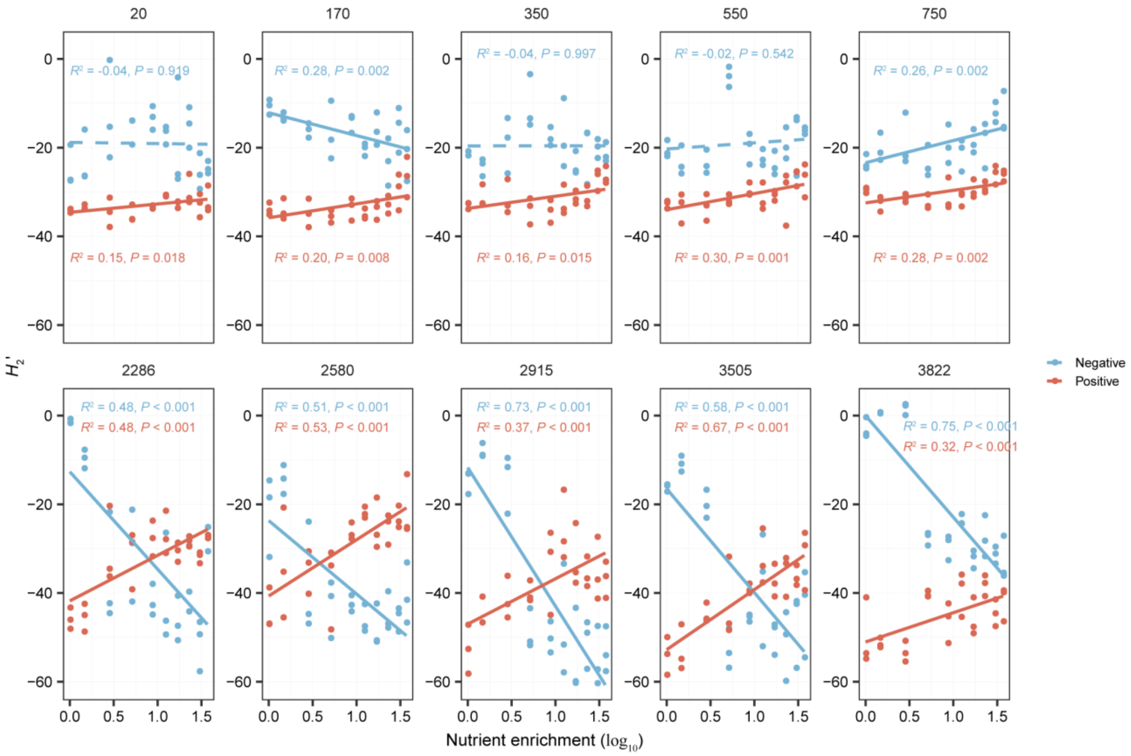

Figure S14. The effect of nutrient enrichment on the specialization H_2' of DOM-bacteria bipartite networks. We plotted the H_2' against the nutrient gradient of nitrate for both negative (blue lines) and positive (red lines) networks for each elevation in China or Norway, and their relationships are indicated by solid ($P \leq 0.05$) or dotted ($P > 0.05$) lines using linear models with one-sided F-statistics.

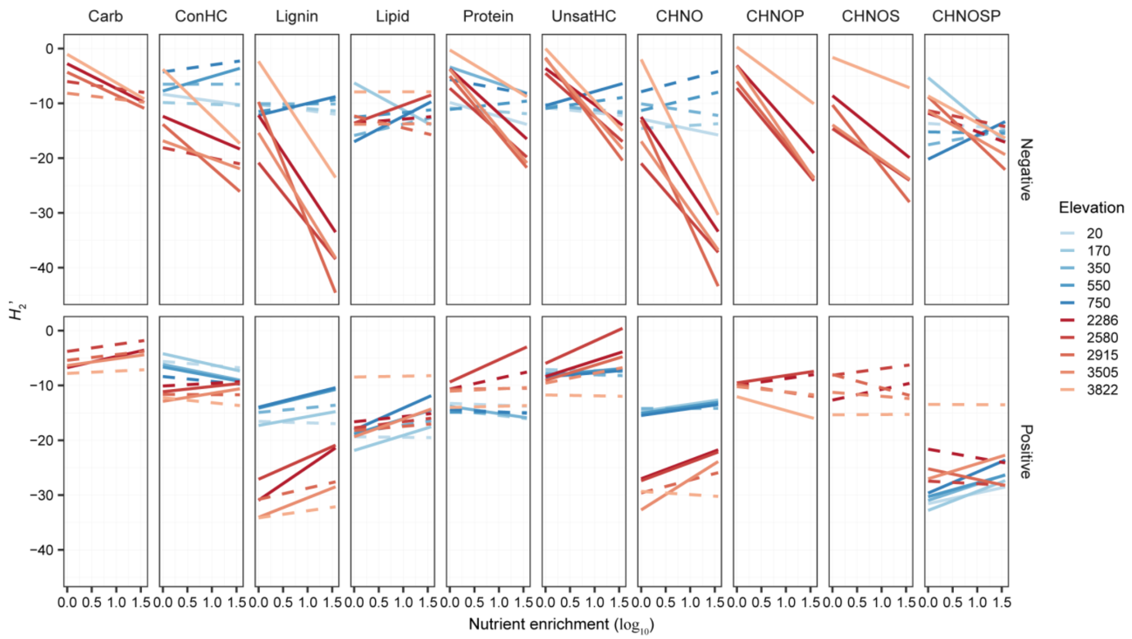

Figure S15. The effects of nutrient enrichment on the specialization H_2' of DOM-bacteria bipartite networks for all formulae and subsets of formulae within the category of compound classes or elemental combinations. We plotted the H_2' against the nutrient gradient of nitrate for both negative (upper panel) and positive (lower panel) networks for each elevation in China (red lines) or Norway (blue lines), and their relationships are indicated by solid ($P \leq 0.05$) or dotted ($P > 0.05$) lines using linear models with one-sided F-statistics.

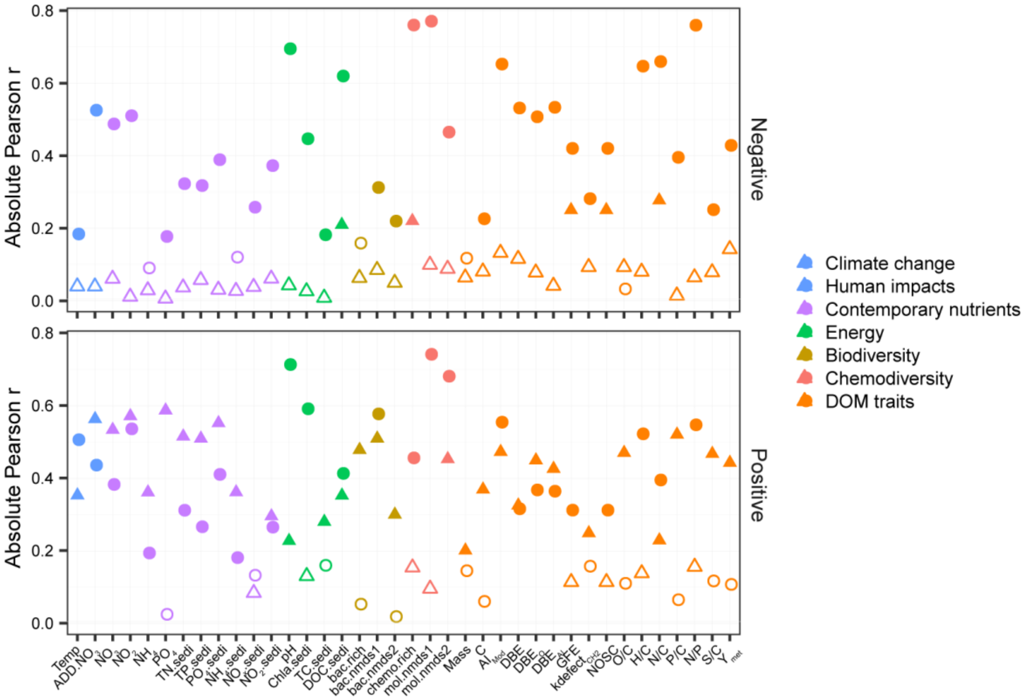

Figure S16. The relative influence of explanatory variables on the specialization H_2' of negative (upper panel) and positive (lower panel) DOM-bacteria bipartite networks using Pearson correlation analysis. Each circle and triangle are the absolute values of Pearson r for individual explanatory variable in China and Norway, respectively. Solid and open circles or triangles indicate the significant ($P \leq 0.05$) and non-significant ($P > 0.05$) two-sided Pearson r, respectively. The details of abbreviations of explanatory variables are available in Table S1.

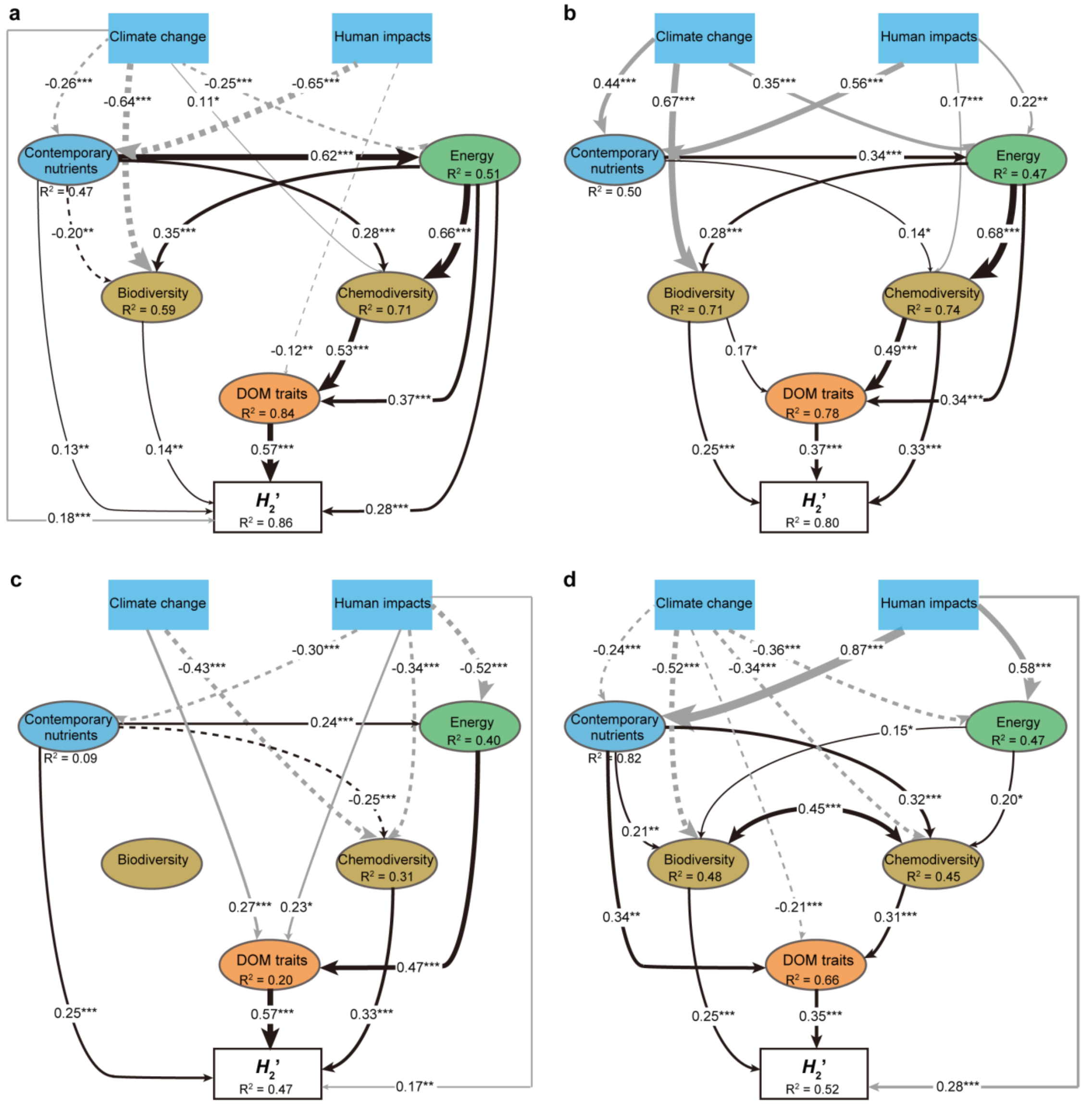

Figure S17. Structural equation models to explain specialization of DOM-bacteria bipartite networks. Best-fitting models illustrate the effects of predictor variables on the H_2' of negative (a, c) and positive (b, d) bipartite networks in China (a-b) or Norway (c-d). Predictor variables were grouped by climate change, human impacts, contemporary nutrients, energy supply, biodiversity, chemodiversity and DOM traits, and described in detail in Table S3. R^2 denotes the proportion of variance explained for the endogenous variables. Dotted and solid arrows indicate the two-sided statistically significantly negative and positive (***, $P \leq 0.001$; **, $P \leq 0.01$; *, $P \leq 0.05$) relationships, respectively, with z-statistics. Grey or black arrows indicate the hypothesized relationships among the

exogenous or endogenous variables and H_2', respectively. Arrow widths and accompanying numbers are the relative effects (that is, standardized path coefficients) of modeled relationships. Composite and observed variables are indicated in ovals and rectangles, respectively. Details of model fit are summarized in Table S4.

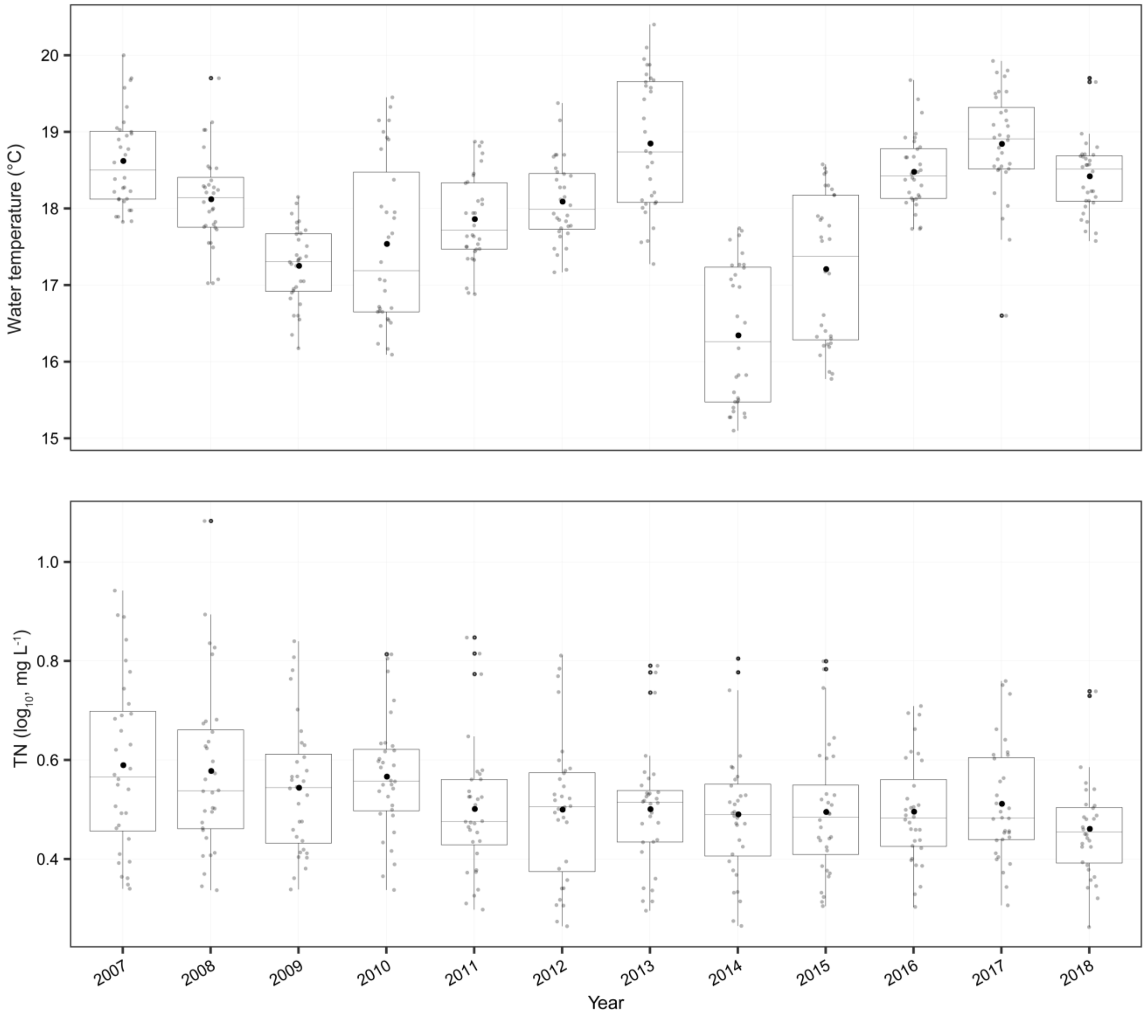

Figure S18. Water temperature and total nitrogen (TN) during 2007-2018 in Taihu Lake. n = 32 sampling sites across the whole of Taihu Lake (Fig. S19a). The grey dots indicate water temperature and TN for individual sites and black dots are the mean values for each year. The boxes extend from 25th to 75th percentile (first and third quartiles), median is marked by the line, and the ends of whiskers indicate the minimum and maximum values within 1.5× the inter-quartile range from the first and third quartiles, respectively.

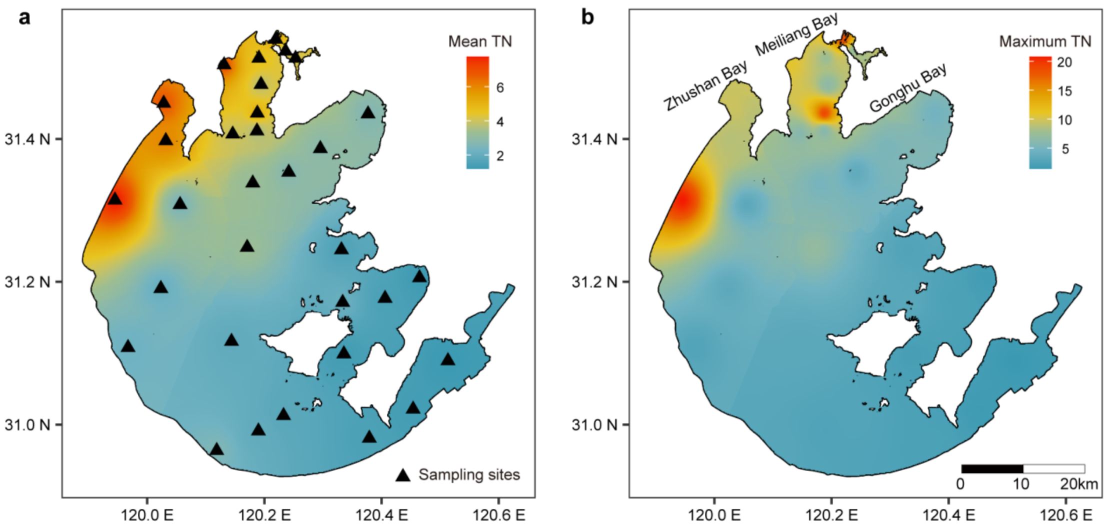

Figure S19. The distribution of mean (a) and maximum (b) total nitrogen (TN) concentrations (mg L^{-1}) in 2007 across the Taihu Lake. Triangles indicate 32 sampling sites.

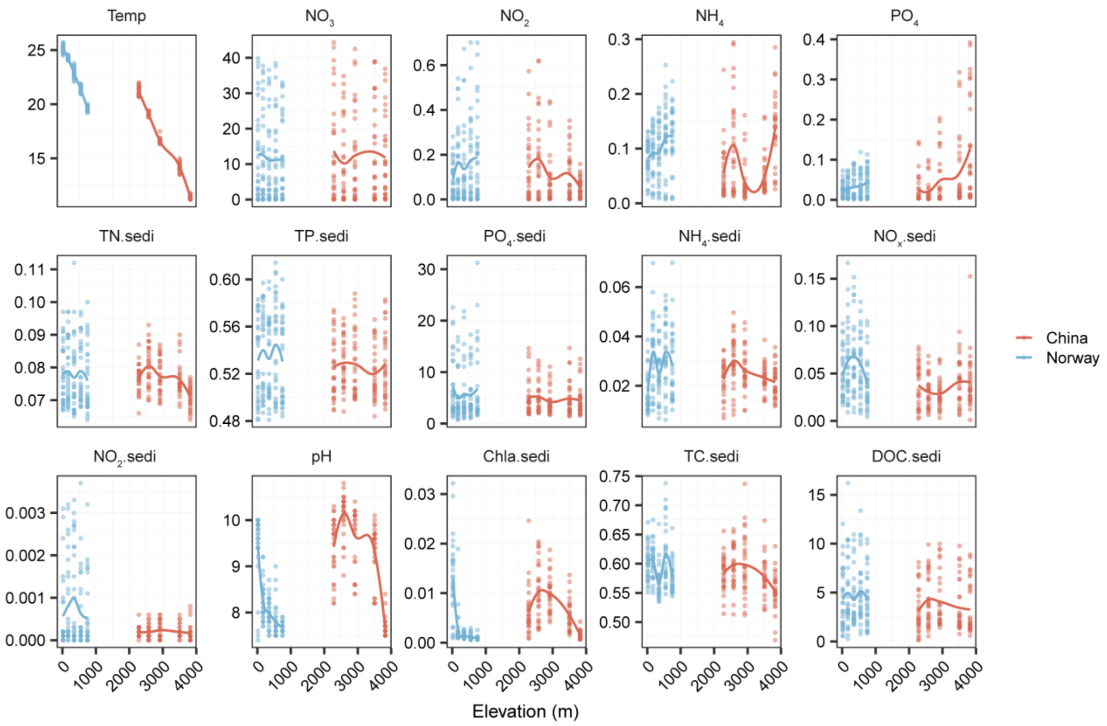

Figure S20. Environmental variables along the elevational gradients, as visualized with loess regression models. The abbreviations of explanatory variables are detailed in Table S1.

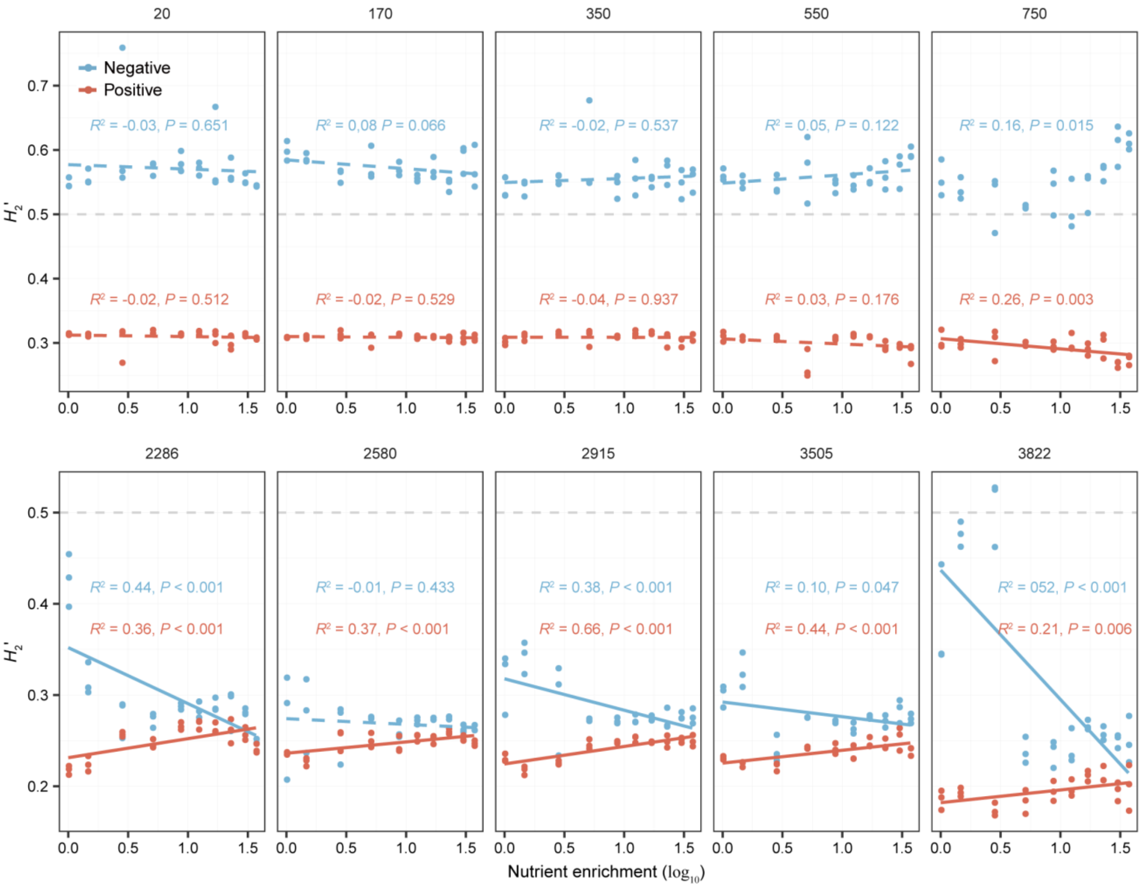

Figure S21. The effects of nutrient enrichment on the observed values of specialization H_2' of DOM-bacteria bipartite networks. We plotted the H_2' against the nutrient gradient of nitrate for both negative (blue lines) and positive (red lines) networks for each elevation in China or Norway, and their relationships are indicated by solid ($P \leq 0.05$) or dotted ($P > 0.05$) lines using linear models with one-sided F-statistics. The horizontal dashed lines indicate more specialized relations with a H_2' value above 0.5.

References

1. de Sassi C, Lewis OT, Tylianakis JM. Plant-mediated and nonadditive effects of two global change drivers on an insect herbivore community. *Ecology* 2012, **93**(8)**:** 1892-1901.

2. Glassman SI, Weihe C, Li J, Albright MBN, Looby CI, Martiny AC*, et al.* Decomposition responses to climate depend on microbial community composition. *Proc Natl Acad Sci U S A* 2018, **115**(47)**:** 11994-11999.

3. Wang J, Pan F, Soininen J, Heino J, Shen J. Nutrient enrichment modifies temperature-biodiversity relationships in large-scale field experiments. *Nat Commun* 2016, **7:** 13960.

4. Jessup CM, Kassen R, Forde SE, Kerr B, Buckling A, Rainey PB*, et al.* Big questions, small worlds: microbial model systems in ecology. *Trends in Ecology & Evolution* 2004, **19**(4)**:** 189-197.
