# Supplementary material for: Ecological networks of dissolved organic matter and microorganisms under global change
Source: Nat Commun. 2022 Jun 23;13:3600. doi: 10.1038/s41467-022-31251-1 (PMC9226077; doi:10.1038/s41467-022-31251-1)
Supplement: Supplementary file 2 — Reporting Summary [file 41467_2022_31251_MOESM2_ESM.pdf]

nature portfolio

Corresponding author(s): Jianjun Wang

Last updated by author(s): May 30, 2022

Reporting Summary

Nature Portfolio wishes to improve the reproducibility of the work that we publish. This form provides structure for consistency and transparency in reporting. For further information on Nature Portfolio policies, see our Editorial Policies and the Editorial Policy Checklist.

Statistics

For all statistical analyses, confirm that the following items are present in the figure legend, table legend, main text, or Methods section.

n/a	Confirmed	
☐	☒	The exact sample size (*n*) for each experimental group/condition, given as a discrete number and unit of measurement
☐	☒	A statement on whether measurements were taken from distinct samples or whether the same sample was measured repeatedly
☐	☒	The statistical test(s) used AND whether they are one- or two-sided<br>*Only common tests should be described solely by name; describe more complex techniques in the Methods section.*
☐	☒	A description of all covariates tested
☐	☒	A description of any assumptions or corrections, such as tests of normality and adjustment for multiple comparisons
☐	☒	A full description of the statistical parameters including central tendency (e.g. means) or other basic estimates (e.g. regression coefficient) AND variation (e.g. standard deviation) or associated estimates of uncertainty (e.g. confidence intervals)
☐	☒	For null hypothesis testing, the test statistic (e.g. *F*, *t*, *r*) with confidence intervals, effect sizes, degrees of freedom and *P* value noted<br>*Give P values as exact values whenever suitable.*
☒	☐	For Bayesian analysis, information on the choice of priors and Markov chain Monte Carlo settings
☒	☐	For hierarchical and complex designs, identification of the appropriate level for tests and full reporting of outcomes
☐	☒	Estimates of effect sizes (e.g. Cohen's *d*, Pearson's *r*), indicating how they were calculated

Our web collection on statistics for biologists contains articles on many of the points above.

Software and code

Policy information about availability of computer code

Data collection	No software was used for data collection.
Data analysis	All statistical analyses and plots were run in Qiime (v1.9), and R statistical software (v3.6.1) with relevant packages such as ftmsRanalysis V1.0.0, vegan V2.4.6, automap V1.0.14, FD V1.0.12, circlize V0.4.10, bipartite V2.15, gradientForest V0.1.17, extendedForest V1.6.1, randomForestSRC V2.8.0, and lavaan V.0.5.23. The DOM profiles were examined with BrukerDaltonik v4.2 and Formularity (v1.0). Details of these softwares are available in the material and methods.

For manuscripts utilizing custom algorithms or software that are central to the research but not yet described in published literature, software must be made available to editors and reviewers. We strongly encourage code deposition in a community repository (e.g. GitHub). See the Nature Portfolio guidelines for submitting code & software for further information.

Data

Policy information about availability of data

All manuscripts must include a data availability statement. This statement should provide the following information, where applicable:

- Accession codes, unique identifiers, or web links for publicly available datasets
- A description of any restrictions on data availability
- For clinical datasets or third party data, please ensure that the statement adheres to our policy

The microbial sequences and meta data generated in this study have been deposited in the MG-RAST database under accession code 17710 (https://www.mg-rast.org/linkin.cgi?project=mgp17710). The monthly monitoring environmental data of Taihu Lake is available under restricted access according to data management policy and the access could be obtained from Taihu Laboratory for Lake Ecosystem Research (http://thl.cern.ac.cn/) or the corresponding author upon

reasonable request. The other data are available under restricted access due to the authors' continuing projects of field experiments on global mountainsides, and the access can be obtained from the corresponding author upon reasonable request.

Field-specific reporting

Please select the one below that is the best fit for your research. If you are not sure, read the appropriate sections before making your selection.

☐ Life sciences ☐ Behavioural & social sciences ☒ Ecological, evolutionary & environmental sciences

For a reference copy of the document with all sections, see nature.com/documents/nr-reporting-summary-flat.pdf

Ecological, evolutionary & environmental sciences study design

All studies must disclose on these points even when the disclosure is negative.

Study description	Macroecological experiments performed in China and Norway by considering two environmental gradients for aquatic microcosms: Temperature and nutrient. We examined sediment DOM compositions and their association with bacteria.
Research sample	There are 300 microcosms for bacterial communities, including 5 elevations from two mountains. For each elevation, ten nutrient levels with three replicates. Such a large number of 300 samples such as 5 elevations and 10 nutrient levels across two mountains enable us to statistically explore the relationships among microbes, DOM and environments.
Sampling strategy	We selected locations with five different elevations on each mountainside. The elevations were 3,822, 3,505, 2,915, 2,580 and 2,286 m a.s.l. on Laojun Mountain in China, and 750, 550, 350, 170 and 20 m a.s.l. on Balggesvarri Mountain in Norway. At each elevation, we established 30 aquatic microcosms (1.5 L bottle) composed of 15 g of sterilised lake sediment and 1.2 L of sterilised artificial lake water, which included one of ten nutrient levels of 0, 0.45, 1.80, 4.05, 7.65, 11.25, 15.75, 21.60, 28.80 and 36.00 mg N L-1 of KNO3. In this case, we had 30 samples at each elevation, and 15 samples at each nutrient levels in each mountain. The number of over 15 samples enabled us to perform necessary statistical analyses for each elevation or nutrient level.
Data collection	For each mountain, we recorded data in situ and tested the samples in lab. The involved instruments included the meters of temperature and pH (Sanxin SX610), Skalar SA1000, illumina Miseq, and 15 Tesla solariX XR system. More details could be found in the main text and in the Wang et al 2016 Nat Commun. The field trip was carried out led by Jianjun Wang.
Timing and spatial scale	The comparative field microcosm experiments were conducted on Laojun Mountain in China (26.6959 N; 99.7759 E) in September-October 2013, and on Balggesvarri Mountain in Norway (69.3809 N; 20.3483 E) in July 2013. We chose these two mountains due to their contrasting climates and maximized the microbial species pools across the regions and elevations.
Data exclusions	We have three replicates for each treatment. There were no data exclusions as we successfully obtained all necessary results for each sample.
Reproducibility	We performed the experiments in the described months in each mountain for one month and applied three replicates for each treatment. All replicates were successful. All details in field setups, laboratory analyses, and data analyses described for reproducibility in the material and methods.
Randomization	Samples were reordered for sampling handling, FT-ICR MS, DNA extraction, PCR amplification and sequencing to rule out potential biases. The randomization is generally not relevant to our study results as our experiments have quality controls in the tests such as with standards in FT-ICR MS.
Blinding	The sample order is independent among the testing of biological, meta data, and DOM analysis. The technicians are essentially having no information about the sample order or the relationships among the samples.

Did the study involve field work? ☒ Yes ☐ No

Field work, collection and transport

Field conditions	The annual temperatures ranged from 4.2-12.9 °C in China and -2.9-0.7 °C in Norway.
Location	Laojun Mountain in China (26.6959 N; 99.7759 E) in September-October 2013, and on Balggesvarri Mountain in Norway (69.3809 N; 20.3483 E) in July 2013.
Access & import/export	This is open field trip and no specific permission is required in respective of academic studies carried out by Nanjing Institute of Geography and Limnology (Chinese Academy of Sciences), and Department of Geosciences and Geography (University of Helsinki).
Disturbance	No specific disturbance was caused due to the small spatial scale needed for field experiments.

Reporting for specific materials, systems and methods

We require information from authors about some types of materials, experimental systems and methods used in many studies. Here, indicate whether each material, system or method listed is relevant to your study. If you are not sure if a list item applies to your research, read the appropriate section before selecting a response.

Materials & experimental systems

n/a	Involved in the study
☒	☐ Antibodies
☒	☐ Eukaryotic cell lines
☒	☐ Palaeontology and archaeology
☒	☐ Animals and other organisms
☒	☐ Human research participants
☒	☐ Clinical data
☒	☐ Dual use research of concern

Methods

n/a	Involved in the study
☒	☐ ChIP-seq
☒	☐ Flow cytometry
☒	☐ MRI-based neuroimaging
